# Bronchodilator response is linked with uncontrolled moderate‐to‐severe childhood asthma and elevated IL‐4 and IL‐13

**DOI:** 10.1111/pai.70392

**Published:** 2026-06-08

**Authors:** Nariman K. A. Metwally, Simone Hashimoto, Susanne J. H. Vijverberg, Anne H. Neerincx, Barbara S. Dierdorp, Tamara Dekker, Eric G. Haarman, Jan Willem Duitman, Mario Gorenjak, Antoaneta A. Toncheva, Susanne Harner, Susanne Brandstetter, Christine Wolff, Paula Corcuera‐Elosegui, Leyre López‐Fernández, Olaia Sardón‐Prado, Maria Pino‐Yanes, Uroš Potočnik, Michael Kabesch, Aletta D. Kraneveld, René Lutter, Suzanne W. J. Terheggen‐Lagro, Mahmoud I. Abdel‐Aziz, Anke H. Maitland‐van der Zee, the SysPharmPediA Consortium

**Affiliations:** ^1^ Department of Pulmonary Medicine, Amsterdam UMC University of Amsterdam Amsterdam the Netherlands; ^2^ Amsterdam Institute for Infection and Immunity, Inflammatory Diseases Amsterdam the Netherlands; ^3^ Amsterdam Public Health, Personalized Medicine Amsterdam The Netherlands; ^4^ Department of Pediatric Pulmonology and Allergy Emma Children's Hospital, Amsterdam UMC Amsterdam The Netherlands; ^5^ Department of Public and Occupational Health, Amsterdam UMC University of Amsterdam Amsterdam the Netherlands; ^6^ Department of Experimental Immunology, Amsterdam UMC University of Amsterdam Amsterdam the Netherlands; ^7^ Center for Human Molecular Genetics and Pharmacogenomics, Faculty of Medicine University of Maribor Maribor Slovenia; ^8^ Department of Pediatric Pneumology and Allergy University Children's Hospital Regensburg (KUNO) Regensburg Germany; ^9^ University Children's Hospital University of Regensburg Regensburg Germany; ^10^ Division of Pediatric Respiratory Medicine Donostia University Hospital San Sebastián Spain; ^11^ Department of Pediatrics. Faculty of Medicine and Nursing University of the Basque Country (UPV/EHU) San Sebastián Spain; ^12^ Genomics and Health Group, Department of Biochemistry, Microbiology, Cell Biology, and Genetics Universidad de La Laguna (ULL) La Laguna Tenerife Spain; ^13^ CIBER de Enfermedades Respiratorias Instituto de Salud Carlos III Madrid Spain; ^14^ Instituto de Tecnologías Biomédicas (ITB) Universidad de La Laguna (ULL) La Laguna Tenerife Spain; ^15^ Faculty of Chemistry and Chemical Engineering University of Maribor Maribor Slovenia; ^16^ Department for Science and Research University Medical Centre Maribor Maribor Slovenia; ^17^ Division of Pharmacology, Utrecht Institute for Pharmaceutical Sciences, Faculty of Science Utrecht University Utrecht CG The Netherlands; ^18^ Department of Genetics, UMC Groningen University of Groningen Groningen Netherlands; ^19^ Department of Clinical Pharmacy, Faculty of Pharmacy Assiut University Assiut Egypt

**Keywords:** asthma control, bronchodilator response, moderate‐to‐severe pediatric asthma, non‐invasive phenotyping, type 2 inflammation

## Abstract

**Background:**

Bronchodilator response (BDR) is a key clinical feature in childhood asthma, but its relation to asthma pathophysiology is not fully understood. This study examines the associations between BDR, disease control, and serum cytokines/chemokines in children with moderate‐to‐severe asthma (MSA).

**Methods:**

BDR was assessed in 140 children (aged 6–17 years, 41% females) from the SysPharmPediA cohort using the ERS/ATS 2022 guidelines (ΔFEV_1_ >10% predicted) and *z*‐score (ΔFEV_1_‐*z*‐score >0.78) post‐bronchodilator definitions. Risk of uncontrolled asthma in relation to BDR was estimated by a logistic regression model, adjusting for baseline lung function, age, sex, BMI *z*‐score, ethnicity, country, season, GINA step, and smoking exposure. Thirty‐nine serum proteins were measured using Luminex Multiplex Assay; levels below the detection limit (LOD) were imputed as LOD/√2, and proteins with >40% missing values across samples were excluded. Children with high and low BDR were compared for serum proteins by linear regression model, adjusting for covariates and applying false discovery rate correction.

**Results:**

Children with high BDR (21%) had significantly higher odds of uncontrolled asthma (adjusted OR ≈ 3.22, 95% CIs: 1.07–11.3) and more frequent severe exacerbations in the past year compared with those with low BDR (70% vs. 46%, *p* < .05). High‐BDR children showed elevated serum levels of IL‐13, IL‐4, TNF‐α, IL‐6, IL‐7, IL‐8, IL‐1β, and MMP‐1 (*q* < 0.05).

**Conclusion:**

In children with MSA, a high BDR is independently associated with poorer asthma control and a distinct systemic inflammatory profile involving T2 and non‐T2 mediators. BDR may serve as a marker for asthma phenotyping.

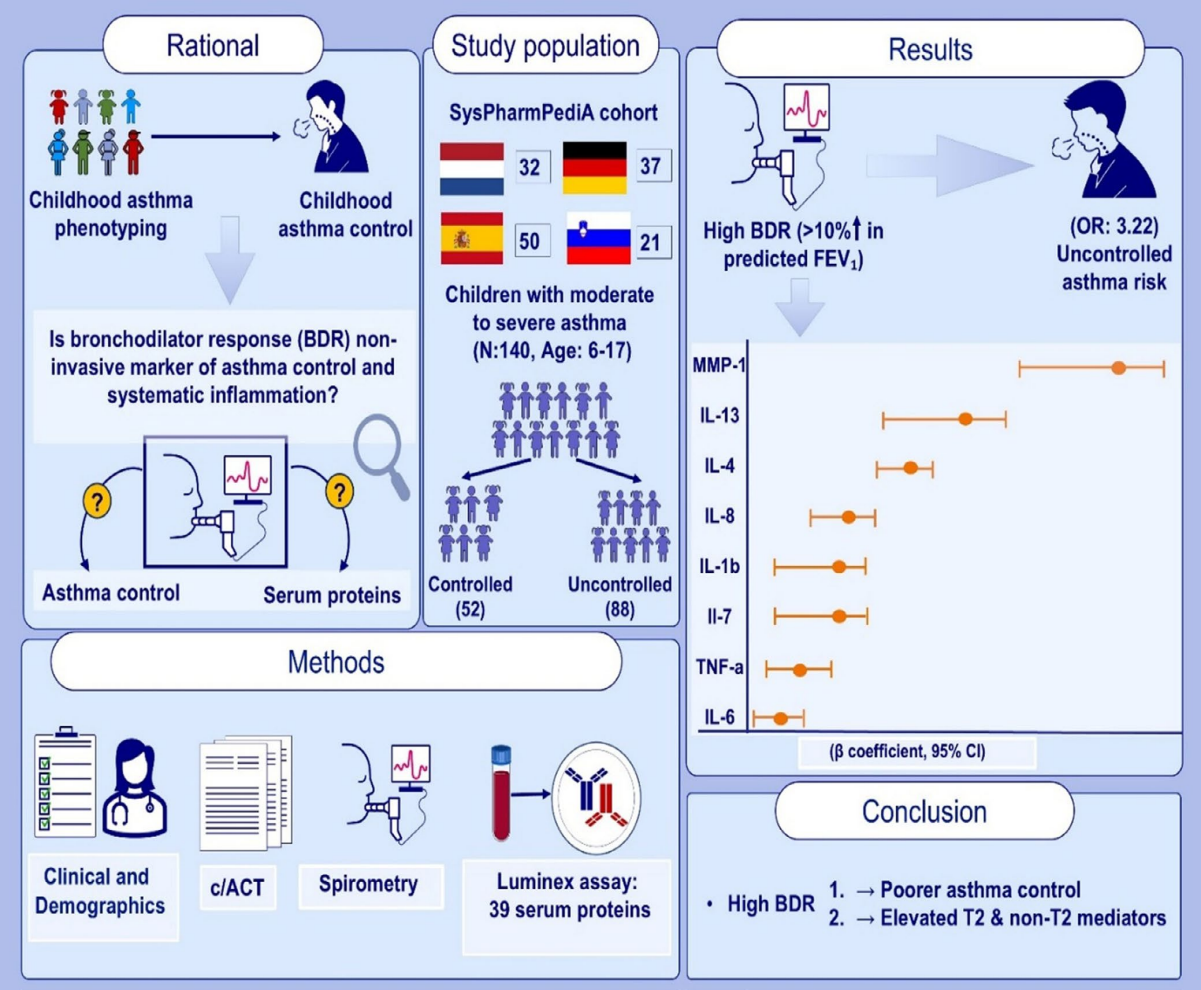

## Graphical abstract

High BDR (≥10% FEV_1_) predicts 3‐fold higher odds of poor asthma control in children with moderate‐to‐severe asthma. High BDR links to elevated T2 cytokines (IL‐4, IL‐13) and non‐T2 mediators (IL‐1β, IL‐6, IL‐7, IL‐8, TNF‐α), as well as MMP‐1. BDR offers non‐invasive phenotyping potential to advance personalized asthma management.

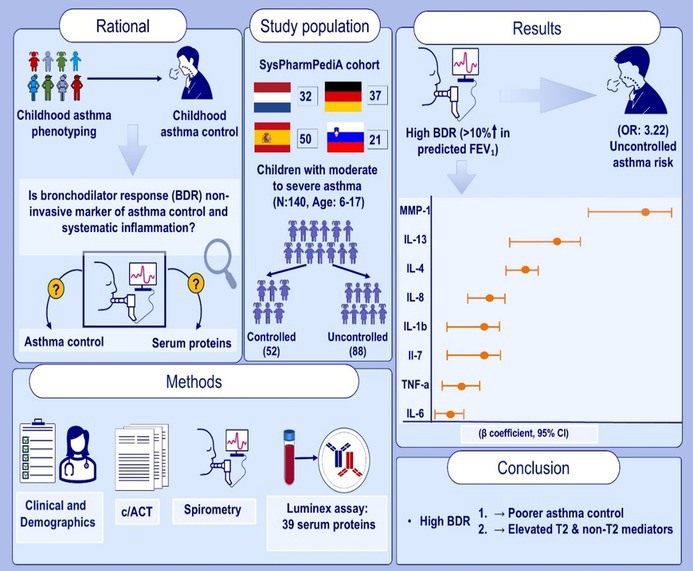

**Key message**

In children with moderate‐to‐severe asthma, a high bronchodilator response serves as a simple, noninvasive marker of poor disease control and a distinct systemic inflammatory signature characterized by elevated IL‐4 and IL‐13, supporting precision phenotyping and targeted treatment strategies.

## 1 INTRODUCTION

Asthma is the most common chronic respiratory condition in children, with moderate‐to‐severe asthma (MSA) representing a smaller yet clinically significant subgroup that poses major challenges.^1^ This subgroup incurs at least twice the healthcare costs, along with higher rates of hospitalization and emergency department visits, and a diminished quality of life compared with children who have less severe disease.^1^ Despite advances in therapeutic management, many children remain poorly controlled, experiencing frequent exacerbations and persistent symptoms,^1^ possibly elevating their long‐term risk of chronic obstructive pulmonary disease (COPD) in adulthood.

This complexity of MSA is further amplified by its multi‐layered heterogeneity, driven by dynamic gene–environment interactions and variable treatment responses.^2^ Simple “one‐size‐fits‐all” categorizations fail to adequately reflect the pathophysiological and clinical diversity of the disease, especially in children. Therefore, accurate phenotyping of pediatric asthma is essential. Conventional phenotyping utilizes the classification of Type 2 (T2)‐high and T2‐low asthma phenotypes, distinguished by levels of T2‐driven airway inflammation. However, not only can children fluctuate between these phenotypes, but many present with overlapping or ambiguous features.^3^ While induced sputum remains the gold standard for direct assessment of airway inflammation, its invasiveness and procedural requirements limit its widespread use in routine pediatric care.^4^, ^5^, ^6^ As an alternative, blood eosinophil and neutrophil counts have been proposed for their accessibility,^6^ but they still require invasive sampling via venipuncture, which can be particularly challenging in children.^7^ Moreover, these markers may not reliably reflect airway‐specific inflammation and are susceptible to extrinsic confounders, including infections and atopic comorbidities, diminishing their diagnostic specificity.^8^, ^9^ Fractional exhaled nitric oxide (FeNO) offers a non‐invasive option for asthma phenotyping and is occasionally used to monitor medication adherence.^10^ However, its clinical value remains debated, as it can be confounded by medication intake, including corticosteroids, which are commonly used in MSA, reducing its reliability in treated patients.^11^

Emerging evidence suggests that bronchodilator response (BDR) may serve as a non‐invasive marker linked to asthma pathophysiology.^12^ BDR, quantified as the percentage increase in forced expiratory volume in 1 s (FEV_1_) after administration of a short‐acting β_2_‐agonist, is a routine component of spirometric evaluation.^12^ While BDR is widely used to support asthma diagnosis, multiple studies indicate that a higher BDR is associated with poorer asthma control in both children and adults.^13^, ^14^ These findings raise the possibility that BDR can contribute to asthma phenotyping. However, previous studies have primarily evaluated BDR in broader asthma populations, while its clinical utility specifically in children with MSA, as well as its relationship with systemic inflammatory profiles, remains insufficiently explored. We hypothesize that BDR may serve as a non‐invasive phenotypic marker in children with MSA and provide additional insights into inflammatory heterogeneity beyond currently available biomarkers. The aims of this study were therefore to (1) assess whether BDR is associated with asthma control and (2) determine whether BDR is linked to distinct systemic inflammatory profiles in children with MSA.

## 2 METHODS

### 2.1 Study design

**FIGURE 1:**
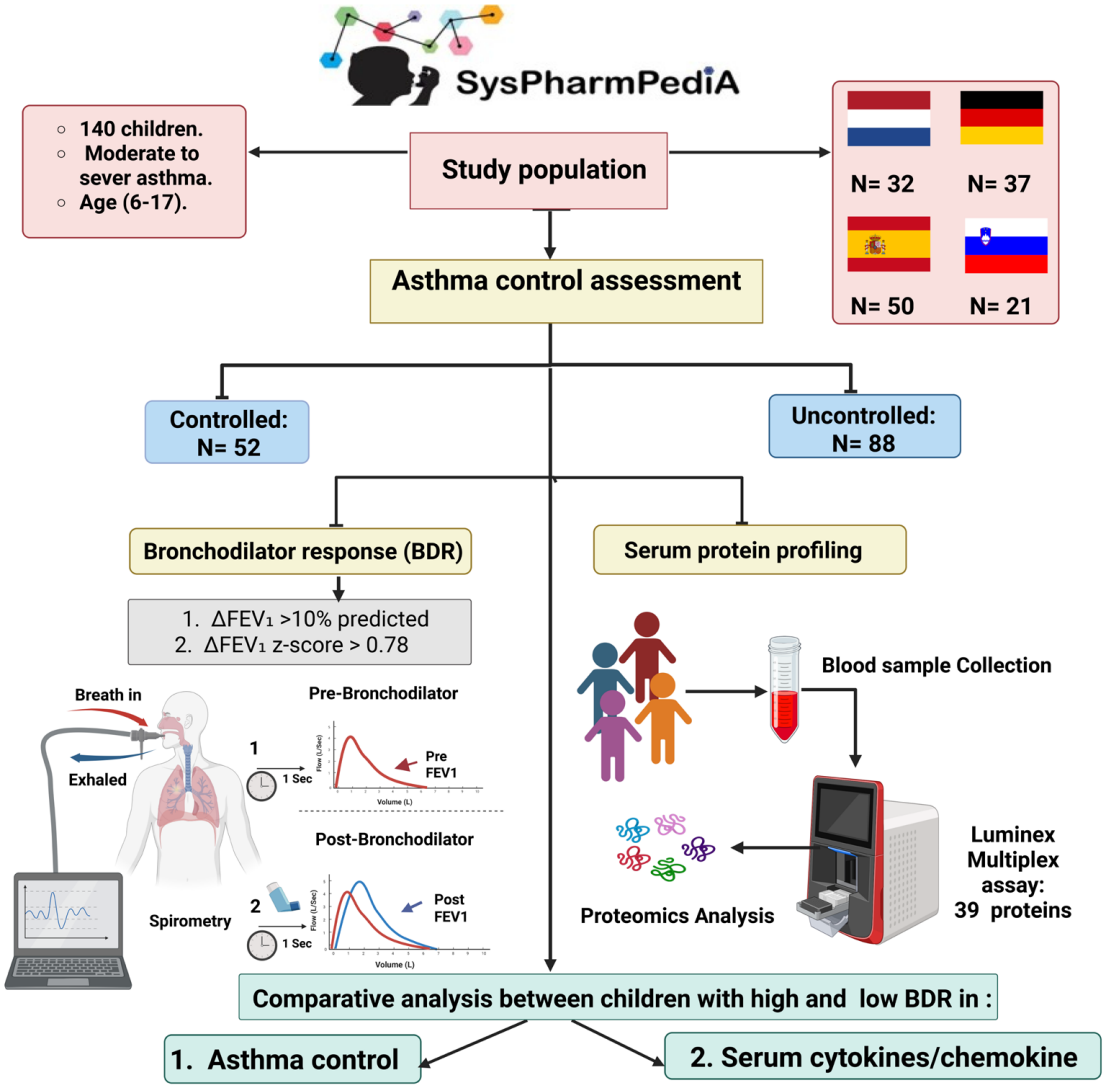
Study Workflow and Methodology. This flowchart outlines the study design, from participant selection to data analysis. All data collection, including asthma control evaluation, blood sampling, and spirometry testing, was performed during patient visits as part of the core SysPharmPediA cohort protocol prior to this analysis. The original cohort included 145 participants, of whom 140 completed spirometry testing and were included in the current study; 5 participants were excluded due to missing spirometry data. Source: Created in BioRender.

A cross‐sectional analysis was performed using data from the SysPharmPediA cohort—an international, multicenter study of 145 children aged 6–17 years with MSA, recruited from the Netherlands, Germany, Slovenia, and Spain. The research was performed in line with the principles of the Declaration of Helsinki and ethics approval was obtained from all participating sites. Informed consent (with assent from children where appropriate) was secured from all participants, and additional cohort details were described previously.^15^ A schematic representation of the study design is shown in Figure 1.

### 2.2 Participant and data collections

Eligibility criteria for the original SysPharmPediA cohort have previously been described in detail at ClinicalTrials.gov (NCT04865575). Briefly, children aged 6–17 years with physician‐confirmed MSA, treated according to Global Initiative for Asthma (GINA) step 3 or higher, were eligible for inclusion. Children were excluded in case of recent antibiotic use within the previous month. In addition, failure to provide written informed consent from parents/caregivers and/or recruited children, when appropriate, resulted in exclusion from study participation. Only children with both baseline and post‐bronchodilator spirometry data were included in the present study (*n* = 140). Study‐specific questionnaires recorded demographic data, medical history, environmental exposures (e.g., smoking), medication use, and self‐reported adherence, which was measured using the validated 5‐item Medication Adherence Report Scale (MARS‐5).^16^ FeNO was measured at 50 mL/s according to ERS/ATS guidelines.^17^ Atopic sensitization (ever) was defined as a positive skin prick test (wheal ≥3 mm) and/or allergen‐specific IgE ≥0.35 kU/L. Peripheral blood was collected for differential leukocyte counts (eosinophils, neutrophils) via fluorescence flow cytometry.^6^ Additional methodological details are available in the Supplementary Material and described previously.^15^

### 2.3 Asthma control assessment

Uncontrolled asthma was defined as current medication use with ≥1 severe exacerbation requiring oral corticosteroids, hospitalization, or emergency room (ER) visit within the past year, or a childhood Asthma Control Test (cACT) or Asthma Control Test (ACT) ≤19.^18^

### 2.4 Lung function and BDR

A total of 140 out of 145 participants performed spirometry. The procedure took place prior to and 15 min post administering 400 μg of inhaled salbutamol, following (ERS/ATS) guidelines.^17^ High BDR was defined as an increase in FEV_1_ >10% of predicted post‐bronchodilator, based on ERS/ATS 2022 criteria.^19^ Additionally, another definition based on *z*‐scores was applied, where a high BDR corresponds to an improvement greater than 0.78 in the post‐bronchodilator FEV_1_ *z*‐score.^20^ Both definitions account for demographic differences in spirometry. A representation of the lung function test measurement using a spirometer is shown in Figure S1.

### 2.5 Serum protein (cytokine and chemokine) analysis

The serum levels of 39 inflammatory mediators were measured using Luminex multiplex immunoassays (R&D Systems Inc., Minneapolis, MN; Invitrogen/ProcartaPlex) with fresh, non‐thawed aliquots, as previously described.^6^, ^21^ For IL‐5, an assay with high sensitivity from R&D Systems Inc. (Minneapolis, MN) was employed for analysis. Data acquisitions were carried out using the Bio‐Plex 200 system (Bio‐Rad, Hercules, CA, USA). Data points with fewer than 25 beads were considered low quality and excluded from further analysis. For analytes below the limit of detection (LOD), imputation by LOD/√2, multiplied by a random factor (0.75–1.25) was performed to minimize skewness in variable distribution, as previously described.^21^ Proteins with >40% missing across all samples were excluded. The full list of the 39 measured inflammatory mediators is provided in Table S1.

### 2.6 Statistical analysis

Comparisons between high and low BDR groups (both BDR definitions) used Mann–Whitney U tests for continuous variables and chi‐square or Fisher's exact tests for categorical data, as appropriate. Logistic regression assessed associations between BDR and risk of uncontrolled asthma, adjusting for baseline FEV_1_, age, sex, BMI *z*‐score, ethnicity, country, season, GINA step, and current smoking exposure (as defined by a Directed Acyclic Graph in Figure S2). Since both BDR definitions already incorporate age, sex, and ethnicity in their calculation, we additionally ran models excluding these variables as covariates to assess the robustness of our findings.

Serum protein levels were compared using Mann–Whitney *U* tests with Benjamini‐Hochberg FDR correction applied for multiple testing. Multiple linear regression models further adjusted for confounders in the DAG to test for differences in protein levels; FDR correction was applied to *p*‐values as well. To explore potential functional relationships between the key cytokines and inflammatory mediators significantly associated with high BDR, we utilized the STRING database to analyze protein–protein interactions (PPI). Pathway enrichment analysis was conducted using the Reactome (REAC) database via g:Profiler on the proteins significantly associated with high BDR group, with FDR <0.05 used to show significantly enriched pathways. All tests were two‐tailed with *α* < 0.05 considered significant. Analyses were completed using R version 4.4.3 and RStudio IDE 2024.12.1, employing the stats, plyr, dplyr, tidyr, reshape2, stringr, ggplot2, ggpubr, and rspiro packages.

## 3 RESULTS

### 3.1 Demographic and clinical characteristics of the study participants

This clinical and demographic information, stratified by BDR status according to the >10% predicted FEV_1_ definition, is summarized in Tables 1, 2, 3. The median age of the participants was 11.97 years (IQR: 9.65–14.00), and 39% were female. Based on the ERS/ATS 2022 definition, 21% (*n* = 30) were classified as having high BDR (>10% increase in predicted FEV_1_). Children with high BDR had significantly poorer asthma control than those with low BDR: 80% had uncontrolled asthma compared to 58% of low BDR children (*p* = .028). Severe asthma exacerbations in the past year were also more frequent in the high BDR group (70% vs. 46%, *p* = .022). Asthma Control Test (ACT) *z*‐scores were lower in the high BDR group, and this difference was statistically significant (median *z*‐score: 0.55 vs. 1.03, *p* = .05).

**TABLE 1:** Demographic and clinical characteristics of children with high and low BDR according to the 10% FEV_1_ definition.

Characteristics	Total (*N* = 140)	Low BDR (ΔFEV_1_ ≤10%) (*n* = 110)	High BDR (ΔFEV_1_ >10%) (*n* = 30)	*p* value
Age in years, median (IQR)	11.97 (9.65, 14.00)	11.52 (9.56, 13.80)	13.35 (10.21, 14.10)	0.164
Sex (Female), *n* (%)	55/140 (39%)	39/102 (38%)	16/38 (42%)	0.677
Ethnicity, *n* (%)				**0.013**
Caucasian	107/139 (77%)	88/109 (81%)	19/30 (63%)	
Latino	10/139 (7%)	9/109 (8%)	1/30 (3%)	
African	6/139 (4%)	4/109 (4%)	2/30 (7%)	
Asian	2/139 (1%)	0/109 (0%)	2/30 (7%)	
Mixed/Others	14/139 (10%)	8/109 (7%)	6/30 (20%)	
BMI *z*‐score, median (IQR)	0.57 (−0.32, 1.38)	0.63 (−0.30, 1.36)	0.47 (−0.48, 1.67)	0.953
Birth and early life factors, *n* (%)				
Mode of delivery (Caesarean section)	26/136 (19%)	21/107 (20%)	5/29 (17%)	0.772
Breastfeeding >4 months	58/134 (43%)	4.00 (0.00, 9.00) (*n* = 105)	4.00 (1.00, 11.00) (*n* = 29)	0.801
Smoking Exposure, *n* (%)				
Smoking exposure during pregnancy	34/127 (27%)	27/102 (26%)	7/25 (28%)	0.877
Current smoke exposure	40/135 (30%)	32/107 (30%)	8/28 (29%)	0.89
Country of Inclusion, *n* (%)				0.098
Spain	50/140 (36%)	43/110 (39%)	7/30 (23%)	
Germany	37/140 (26%)	30/110 (27%)	7/30 (23%)	
The Netherlands	32/140 (23%)	20/110 (18%)	12/30 (40%)	
Slovenia	21/140 (15%)	17/110 (15%)	4/30 (13%)	
Inclusion season, *n* (%)				0.06
Winter	27/140 (19%)	23/110 (21%)	4/30 (13%)	
Spring	38/140 (27%)	33/110 (30%)	5/30 (17%)	
Summer	46/140 (33%)	30/110 (27%)	16/30 (53%)	
Autumn	29/140 (21%)	24/110 (22%)	5/30 (17%)	
Clinical Status, *n* (%)				
Uncontrolled Asthma^a^	88/140 (63%)	64/110 (58%)	24/30 (80%)	**0.028**
(Childhood) Asthma Control Test ((c) ACT) *z*‐score, median (IQR)	0.95 (0.30, 1.55) (*n* = 138)	1.03 (0.30, 1.55) (*n* = 109)	0.55 (0.12, 1.03) (*n* = 29)	**0.05**
Severe asthma exacerbations in the past year	72/140 (51%)	51/110 (46%)	21/30 (70%)	**0.022**

*Note*: Demographic, and clinical characteristics are presented for the total cohort and stratified by BDR status according to the >10% increase in predicted FEV_1_ definition (high BDR). The low BDR group comprised children who did not meet the respective BDR criterion. Continuous variables are presented as medians with interquartile ranges, while categorical variables are shown as counts and percentages. Comparisons between high BDR and low BDR groups were conducted using the Mann–Whitney *U* test for continuous data and either the chi‐squared test or Fisher's exact test for categorical data, as appropriate. A *p*‐value below 0.05 showed evidence of a significant difference between groups and is highlighted in boldface.

Abbreviations: BMI, body mass inded; IQR, interquartile range.

^a^ Uncontrolled asthma is characterized by a (childhood) Asthma Control Test (cACT) score of 19 or below and/or the occurrence of severe exacerbations within the previous year that necessitated hospitalization, emergency room visits, or the use of oral corticosteroids (OCS).

**TABLE 2:** Lung function, FeNO, white blood cell counts, and atopic sensitization in children with high and low BDR according to the 10% FEV_1_ definition.

Characteristics	Total (*N* = 140)	Low BDR (ΔFEV_1_ ≤10%) (*n* = 110)	High BDR (ΔFEV_1_ >10%) (*n* = 30)	*p* value
Lung function				
FEV_1_ pre‐salbutamol % predicted, median (IQR)	94.88 (82.68, 103.29)	96.47 (86.12, 104.44)	85.14 (79.83, 94.84)	**0.002**
FEV_1_ post‐salbutamol % predicted, median (IQR)	99.55 (89.97, 108.76)	99.66 (89.44, 108.16)	99.08 (95.81, 109.95)	0.631
FEV_1_ pre‐salbutamol *z*‐score, median (IQR)	−0.42 (−1.45, 0.28)	−0.30 (−1.18, 0.37)	−1.25 (−1.68, −0.45)	**0.002**
FEV_1_ post‐salbutamol *z*‐score, median (IQR)	−0.04 (−0.84, 0.73)	−0.03 (−0.92, 0.71)	−0.08 (−0.36, 0.84)	0.633
FeNO (ppb), median (IQR)	16.00 (8.83, 36.90) (*n* = 122)	14.00 (8.15, 33.92) (*n* = 98)	18.80 (10.38, 46.83) (*n* = 24)	0.244
White blood cell count (×10^9^/L), median (IQR)				
Eosinophil	0.37 (0.21, 0.64) (*n* = 123)	0.37 (0.20, 0.62) (*n* = 97)	0.36 (0.24, 0.66) (*n* = 26)	0.546
Neutrophil	3.24 (2.42, 4.20) (*n* = 123)	3.20 (2.43, 4.12) (*n* = 97)	3.29 (2.33, 4.42) (*n* = 26)	0.66
Lymphocyte	2.58 (2.23, 3.10) (*n* = 123)	2.57 (2.23, 3.10) (*n* = 97)	2.62 (2.11, 3.10) (*n* = 26)	0.68
Eosinophilia, *n* (%)				
>0.3 × 10^9^/L	78/123 (63%)	61/97 (63%)	17/26 (65%)	0.814
>0.5 × 10^9^/L	44/123 (36%)	35/97 (36%)	9/26 (35%)	0.89
Atopy and allergic sensitization, *n* (%)^a^				
Atopy	119/134 (89%)	93/105 (89%)	26/29 (90%)	1
Aeroallergen combined	119/136 (88%)	94/107 (88%)	25/29 (86%)	0.759
HDM	95/133 (71%)	75/104 (72%)	20/29 (69%)	0.74
Grass pollen	75/133 (56%)	57/104 (55%)	18/29 (62%)	0.486
Mold	13/110 (12%)	8/86 (9%)	5/24 (21%)	0.152
Cat	46/125 (37%)	33/100 (33%)	13/25 (52%)	0.078
Dog	38/119 (32%)	24/96 (25%)	14/23 (61%)	**0.001**
IgE, abnormal	98/113 (87%)	74/86 (86%)	24/27 (89%)	1
Allergic rhinitis (ever), *n* (%)	101/133 (76%)	82/104 (79%)	19/29 (66%)	0.138
Atopic dermatitis (Eczema)	52/128 (41%)	40/100 (40%)	12/28 (43%)	0.786

*Note*: Lung function, FeNO, white blood cell counts, and atopy characteristics are presented for the total cohort and stratified by BDR status according to the >10% increase in predicted FEV_1_ definition (high BDR). The low BDR group comprised children who did not meet the respective BDR criterion. Continuous variables are presented as medians with interquartile ranges, while categorical variables are shown as counts and percentages. Comparisons between high BDR and low BDR groups were conducted using the Mann–Whitney *U* test for continuous data and either the chi‐squared test or Fisher's exact test for categorical data, as appropriate. A *p*‐value below 0.05 showed evidence of a significant difference between groups. Boldface values indicate statistically significant differences of *p*‐values *p* < (0.05).

Abbreviations: FEV_1_, forced expiratory volume in 1 second; FeNO, fractional exhaled nitric oxide; IQR, interquartile range.

^a^ Atopic sensitization is defined based on a physician‐documented history of sensitization to airborne allergens, demonstrated by a positive skin prick test (wheal diameter ≥3 mm) and/or elevated allergen‐specific IgE levels (≥0.35 kU/L).

**TABLE 3:** Asthma medication use and treatment steps in children with high and low BDR according to the 10% FEV_1_ definition.

Characteristics	Total (*N* = 140)	Low BDR (ΔFEV_1_ ≤10%) (*n* = 110)	High BDR (ΔFEV_1_ >10%) (*n* = 30)	*p* value
Asthma medication *n* (%)				
ICS	140 (100%)	110 (100%)	30 (100%)	N/A
SABA	130/140 (93%)	102/110 (93%)	28/30 (93%)	1
LABA	132/140 (94%)	105/110 (95%)	27/30 (90%)	0.368
OCS	4/140 (3%)	2/110 (2%)	2/30 (7%)	0.201
LTRA	25/140 (18%)	16/110 (15%)	9/30 (30%)	**0.05**
Anticholinergics	17/140 (12%)	15/110 (14%)	2/30 (7%)	0.527
Biologics (Omalizumab/Mepolizumab)	15/140 (11%)	9/110 (8%)	6/30 (20%)	0.091
GINA Steps, *n* (%)				0.15
Step 3 (GINA)	63/140 (45%)	53/110 (48%)	10/30 (33%)	
Step 4 (GINA)	61/140 (44%)	47/110 (43%)	14/30 (47%)	
Step 5 (GINA)	16/140 (11%)	10/110 (9%)	6/30 (20%)	
MARS‐5 (≥21), *n* (%)	117/126 (93%)	95/101 (94%)	22/25 (88%)	0.38

*Note*: Treatment characteristics are presented for the total cohort and stratified by BDR status according to the >10% increase in predicted FEV_1_ definition (high BDR). The low BDR group comprised children who did not meet the respective BDR criterion. Continuous variables are presented as medians with interquartile ranges, while categorical variables are shown as counts and percentages. Comparisons between high BDR and low BDR groups were conducted using the Mann–Whitney *U* test for continuous data and either the chi‐squared test or Fisher's exact test for categorical data, as appropriate. A *p*‐value below 0.05 showed evidence of a significant difference between groups and is highlighted in boldface.

Abberviations: GINA, global initiative for asthma; ICS, inhaled corticosteroid; IQR, interquartile range; LABA, long‐acting β‐agonist; LTRA, leukotriene antagonist; MARS‐5, Medication Adherence Report Scale‐5; OCS, oral corticosteroid; SABA, short‐acting β‐agonist.

Ethnic differences were noted, with mixed/other ethnicities overrepresented among high BDR children (20% vs. 7%), while Caucasian children were more prevalent in the low BDR group (63% vs. 81%, *p* < .05). Sensitization to aeroallergens was also more common in high BDR children: sensitization to dog dander (61% vs. 25%, *p* = .001) and, to a lesser extent and not statistically significant, cat dander (52% vs. 33%, *p* = .078).

Baseline lung function was markedly reduced in high BDR children, with a median pre‐bronchodilator FEV_1_ % predicted of 85.1 compared to 96.5 in low BDR children (*p* < .001). Post‐bronchodilator FEV_1_ did not differ significantly between groups. No differences were observed for blood eosinophil (at 0.3 × 10^9^/L or 0.5 × 10^9^/L cut‐offs) or neutrophil counts or for FeNO. All children were treated with inhaled corticosteroids. Use of biologics (omalizumab or mepolizumab) tended to be higher among high BDR children (20% vs. 8%, *p* = .091), as was the use of leukotriene receptor antagonists (LTRA) (30% vs. 15%, *p* = .05). Medication adherence was high across all groups (≥93%), with no significant differences between high and low BDR children.

When applying the alternative *z*‐score definition (ΔFEV_1_ *z*‐score >0.78), 27% of children (*n* = 38) were classified as having high BDR (Figure S3). Findings were consistent with those observed for the 10% FEV_1_ definition, with additional significant differences identified in the high BDR group, specifically higher rates of cat sensitization and use of biologics (Table S2).

### 3.2 Association of BDR with asthma control

Multivariable logistic regression showed that children with high BDR (>10%) had significantly higher odds of uncontrolled asthma compared to those with low BDR, after adjustment for covariates (OR: 3.22; 95% CI: 1.07–11.3; *p* = .048) (Table 4). GINA treatment step was also strongly associated with asthma control: children at step 5 were associated with increased odds of uncontrolled asthma compared to those at step 3 (OR: 16.2; 95% CI: 2.22–343; *p* = .018), and step 4 also showed a significant association (OR: 2.69; 95% CI: 1.03–7.33; *p* = .046). Other covariates, including baseline FEV_1_, age, sex, BMI *z*‐score, ethnicity, country, inclusion season, and smoking exposure, were not predictive of asthma control. Analyses using the *z*‐score definition showed a similar pattern as the 10% FEV_1_ definition (Table S3). Comparable results were also obtained using two definitions when models were adjusted for a reduced set of covariates excluding age, sex, and ethnicity (Tables S4 and S5).

**TABLE 4:** Multivariate logistic regression model showing the association between high BDR according to the FEV_1_ >10% definition and asthma control after adjusting for confounders.

Characteristic (*n* = 134)	OR	SE	95% CI	*p*‐value
BDR (FEV_1_ >10% of predicted)	3.22	0.591	1.07, 11.3	**0.048**
Baseline FEV_1_ (L)	1.09	0.510	0.40, 2.99	0.870
Age	0.94	0.135	0.72, 1.22	0.630
Sex (male)	0.80	0.452	0.33, 1.94	0.620
BMI (*Z*‐score)	0.99	0.178	0.70, 1.41	0.971
Ethnicity (other/mixed)	2.41	0.669	0.69, 10.0	0.188
Country of inclusion^a^				
Netherlands	1.53	0.743	0.36, 6.76	0.566
Slovenia	1.56	0.706	0.39, 6.34	0.527
Spain	2.38	0.583	0.76, 7.66	0.137
Inclusion season^b^				
Spring	0.83	0.582	0.26, 2.59	0.751
Summer	0.53	0.657	0.14, 1.88	0.337
Winter	1.08	0.672	0.29, 4.14	0.906
GINA treatments step^c^				
Step4	2.69	0.496	1.03, 7.33	**0.046**
Step5	16.2	1.18	2.22, 343	**0.018**
Current smoking exposure	0.81	0.471	0.32, 2.06	0.657

*Note*: Multivariate logistic regression model showing the association between high bronchodilator response (BDR) according to the FEV_1_ >10% definition and asthma control after adjusting for different confounders. Odds ratios (ORs) and their 95% confidence intervals (CIs) for uncontrolled asthma are shown for each variable, with controlled asthma as the reference group. Variables include bronchodilator response, defined as >10% increase in predicted FEV_1_ (high_1_ BDR), baseline FEV_1_, age, sex, ethnicity, country of inclusion, BMI *z*‐score, season of inclusion, GINA treatment step, and current smoking exposure. A *p*‐value < .05 was considered statistically significant and is highlighted in boldface.

Abbreviations: BDR, bronchodilator response; BMI, body mass index; CI, confidence interval; FEV_1_, forced expiratory volume in one second; GINA, Global Initiative for Asthma; OR, odds ratio; SE, standard error.

^a^ Germany was selected randomly as a reference group for comparison of country of inclusion.

^b^ Autumn was selected randomly as a reference group for comparison of inclusion season.

^c^ Step 3 was selected as a reference group for comparison of GINA treatment.

### 3.3 Association of BDR with serum inflammatory markers

Children with high BDR (>10% FEV_1_) demonstrated significantly higher serum levels of interleukin (IL)‐4, IL‐13, tumor necrosis factor‐α (TNF‐α), matrix metalloprotease (MMP‐1), IL‐6, IL‐7, monocyte chemoattractant protein‐4 (MCP‐4, CCL13) thymus and activation regulated chemokine (TARC, CCL17), and IL‐1β compared to children with low BDR. After correction for multiple comparisons using FDR, IL‐4, IL‐13, TNF‐α, and MMP‐1 remained significantly elevated (*q* < 0.05).

**FIGURE 2:**
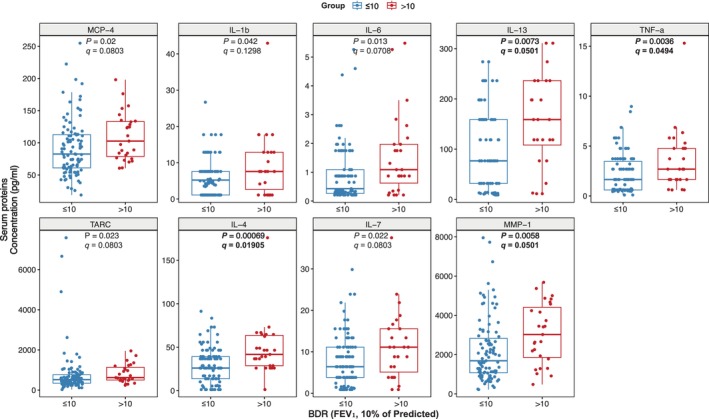
Boxplots of serum protein levels of inflammatory markers in children with high BDR and low BDR, shown separately for BDR definition: >10% increase in predicted FEV_1_ (high BDR). Boxplots display the distribution of significantly different cytokines and proteins between groups. Blue boxes represent the low BDR group, while red boxes represent the high BDR group. BDR, bronchodilator response; FEV_1_, forced expiratory volume in 1 s; CRP, C‐reactive protein; IL, interleukin; MCP, monocyte chemoattractant protein; MMP, matrix metalloproteinase; TARC, thymus and activation regulated chemokine; TNF‐α, tumor necrosis factor alpha. A statistically significant q‐value < .05 is highlighted in boldface.

**FIGURE 3:**
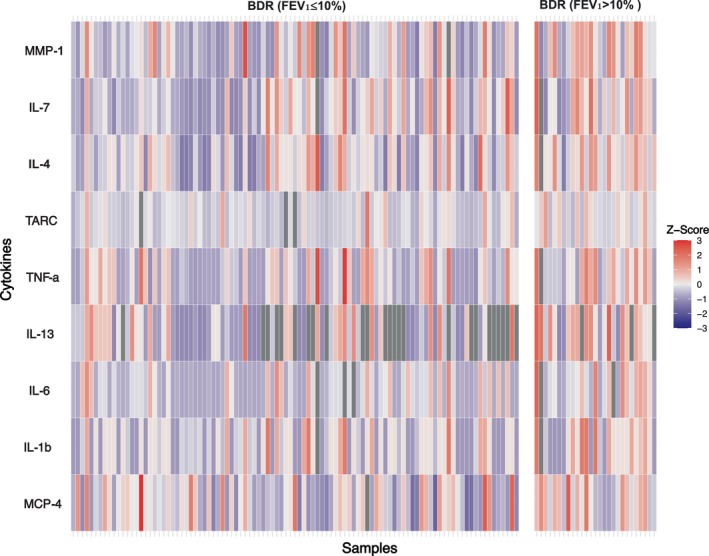
Heatmaps of serum cytokine and chemokine profiles in children with high and low BDR shown for BDR >10% increase in predicted FEV_1_ (high BDR). Each row represents a cytokine or protein, and each column represents an individual participant. Color intensity reflects relative expression levels, illustrating the separation between high BDR and low BDR groups; CRP = C‐reactive protein; IL, interleukin; MCP, monocyte chemoattractant protein; MMP, matrix metalloproteinase; TARC, thymus and activation regulated chemokine; TNF‐α, tumor necrosis factor alpha.

These findings are illustrated in Figure 2, which presents boxplots of cytokine and chemokine levels of inflammatory mediators, and in Figure 3, where heatmaps demonstrate distinct separation between high and low BDR groups based on the abundance of these inflammatory mediators. For the z‐score definition and following FDR correction, additional proteins to the previous proteins, IL‐6, IL‐7, and IL‐10 had significantly higher serum levels in children with high BDR (all *q* < 0.05) (Figures S4 and S5).

**FIGURE 4:**
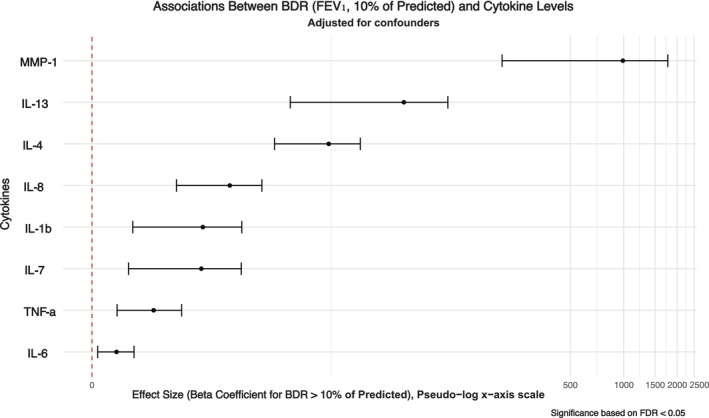
Adjusted associations between high bronchodilator response (BDR) and serum protein levels. Associations for high BDR defined as >10% increase in predicted FEV_1_. Beta coefficients (*β*) represent the direction and magnitude of association, with FDR‐adjusted *p*‐values indicating statistical significance. *β* coefficients are displayed as point estimates, with horizontal lines representing the corresponding 95% confidence intervals. All models were adjusted for age, sex, ethnicity, BMI *z*‐score, GINA treatment step, baseline lung function, study site, season of inclusion, and current smoking exposure. FEV_1_, forced expiratory volume in 1 s; BDR, bronchodilator response, IL, interleukin, TNF‐α, tumor necrosis factor alpha, MMP, matrix metalloproteinase, FDR, false discovery rate. A pseudo‐logarithmic *x*‐axis was used only for visualization purposes to allow plotting cytokines in different pg/mL ranges in one plot.

After adjustment for potential confounders and FDR correction, high BDR (>10% FEV_1_) remained significantly associated with elevated serum levels of multiple inflammatory mediators in the multiple linear regression models. The strongest associations were observed for MMP‐1, IL‐13, and IL‐4, with additional significant findings for IL‐7, IL‐8, IL‐6, IL‐1β, and TNF‐α (Figure 4). Results based on the z‐score definition were largely consistent, although IL‐10 additionally emerged as significant, whereas MMP‐1 was no longer associated (Figure S6).

STRING analysis of the significantly elevated proteins in children with high BDR revealed an interconnected network, and these proteins likely contribute to overlapping signaling pathways related to immune activation and cytokine‐mediated airway inflammation, suggesting functional associations among these inflammatory mediators (Figure S7).

### 3.4 Pathway enrichment analysis

Pathway enrichment analysis for the high BDR enriched proteins (MMP‐1, IL‐4, IL‐13, IL‐6, IL‐7, IL‐8, TNF, IL‐10, and IL‐1β) revealed strong enrichment for several immune‐related pathways, with the most significant being IL‐4 and IL‐13 signaling (*p*adj = 2.20 × 10^−13^), and IL‐10 signaling (*p*adj = 3.27 × 10^−8^) (Figure S8).

## 4 DISCUSSION

This study provides novel evidence that BDR, a widely used, non‐invasive measure of airflow reversibility, can serve as a meaningful phenotypic marker of disease burden and systemic inflammation in children with MSA. Our findings demonstrate that children with high BDR, despite inhaled corticosteroid treatment, are more likely to have uncontrolled asthma and a systemic inflammatory profile characterized by elevations in both T2 and non‐T2 inflammatory mediators. Taken together, these results support the use of BDR as more than a physiologic diagnostic tool, revealing its potential role in pediatric asthma phenotyping and personalized treatment strategies.

Our analyses demonstrated that high BDR is an independent predictor of uncontrolled asthma, among children with MSA exhibiting approximately a threefold increased risk of poor asthma control after adjusting for multiple confounders. The association persisted across additional analyses that excluded demographic covariates already accounted for in BDR reference equations, confirming the robustness of the observed relationship.

Importantly, high BDR may reflect improvement arising from an underlying deficit in airway function rather than a superior therapeutic response. This indicates significant airway hyper‐responsiveness characterized by exaggerated airway narrowing and instability. Accordingly, the ‘room for improvement’ signaled by a high BDR serves as a marker of airway hyper‐responsiveness, conferring increased risk of uncontrolled asthma and exacerbations despite bronchodilator responsiveness.

Although children with high BDR exhibited lower baseline FEV_1_, their post‐bronchodilator values were normalized, consistent with a reversible airflow obstruction pattern. However, FEV_1_ itself did not independently predict uncontrolled asthma in the adjusted model. This contrast highlights that BDR provides additional prognostic value as a dynamic measure of airway responsiveness rather than a static marker of airflow limitation.

Medication patterns reinforced the clinical relevance of high BDR. While all children received ICS and exhibited similar use of short‐ and long‐acting β_2_‐agonists, those with high BDR were more likely to be treated with LTRA and biologic therapies. This pattern reflects more severe disease and the need for controller therapy in children exhibiting high BDR. Notably MARS‐5 adherence was high across groups as only compliant children were included in this study by design,^22^ suggesting treatment differences are unlikely due to poor adherence.

Our results align with previous pediatric data. Galant et al. demonstrated that high BDR phenotype (≥10%–12% FEV_1_), even in untreated children with otherwise normal baseline spirometry, was strongly associated with poor asthma control, atopy, nocturnal symptoms, and increased β_2_‐agonist use.^23^ Similarly, Coverstone et al. (2019) linked BDR with severe asthma, reduced lung function, and higher healthcare utilization.^13^ In contrast to our findings, Coverstone et al. (2019) observed elevated FeNO levels in children with high BDR^13^; however, FeNO levels did not significantly differ between BDR groups in our cohort. This difference might reflect variability in ICS use or adherence, as ICS therapy is known to reduce airway inflammation and FeNO levels, which is consistent with the high treatment adherence reported in our study.^13^, ^24^

Notably, similar patterns have been observed in adult asthma populations. Godoy Fernandes et al., Ferrer Galván et al., and Heffler et al. showed that persistent high BDR, even with treatment, predicted poor asthma control and future risk.^14^, ^25^, ^26^ More recently, Kaminsky et al. examined BDR across diverse study populations and found no association with overall asthma control or symptom burden.^27^ However, their pediatric subgroup analysis revealed that higher BDR correlated with increased symptom burden, underscoring the importance of pediatric‐specific research since findings from mixed‐age cohorts may not fully apply to children with asthma.^19^, ^27^

Interestingly, in our study, children with high BDR demonstrated disproportionately high sensitization to dog and cat allergens. This is in line with previous pediatric studies linking sensitization to dog and cat allergens with greater asthma severity, impaired pulmonary function, and increased symptom burden.^28^, ^29^ Persistent exposure to pet allergens may contribute to chronic airway inflammation and airway hyperresponsiveness, potentially reinforcing the association between allergen sensitization and elevated BDR observed in our cohort.

Ethnic disparities were also evident, with mixed and non‐Caucasian children overrepresented in the high BDR group. This observation is consistent with prior evidence demonstrating greater asthma severity among non‐Caucasian populations, driven by a complex interplay of social determinants, including environmental exposures, housing conditions, allergen burden, and healthcare access, as well as genetic susceptibilities.^19^, ^27^, ^30^ Importantly, genetic factors linked to ethnicity may independently influence asthma outcomes or interact with socioeconomic variables to modulate disease severity.^30^

One of the most novel and impactful findings of our analysis is the characterization of systemic inflammatory profiles in relation to BDR. High BDR was linked to significantly elevated levels of classical type 2 cytokines, including IL‐4 and IL‐13. This pattern reflects type 2 airway inflammation, which is associated with frequent exacerbations and poor asthma control.^31^ IL‐4 and IL‐13 are key mediators of T2 airway inflammation and are associated with airway hyperresponsiveness, mucus overproduction, and structural remodeling in asthma.^31^

Importantly, our data also revealed significant elevations in key pleiotropic pro‐inflammatory cytokines including IL‐6, IL‐1β, and TNF‐α. These cytokines, characteristic of non‐T2 inflammation^32^, ^33^ are known to drive neutrophilic recruitment, tissue injury, airway remodeling, and steroid resistance.^34^, ^35^ Notably, IL‐1β, TNF‐α, and IL‐6 may contribute to both non‐T2 and T2 inflammatory responses, further highlighting the complexity of inflammatory pathways associated with high BDR.^36^, ^37^, ^38^ Elevated serum IL‐6 has been associated with a 24% increased odds of corticosteroid‐requiring exacerbations and more asthma severity symptoms.^39^ Moreover, elevated IL‐6 levels have been implicated in asthma exacerbations characterized by overlapping eosinophilic and neutrophilic inflammation, which are often associated with poorer pulmonary function relative to other inflammatory phenotypes.^33^, ^40^ Collectively, these complex cytokine interactions contribute to asthma heterogeneity and treatment responsiveness.^33^, ^35^

In addition, IL‐8 and IL‐7 were significantly elevated in children with high BDR. Elevated IL‐8 (CXCL8) levels have been linked to neutrophilic airway inflammation, airway remodeling, disease severity, and corticosteroid resistance in asthma.^33^ On the other hand, IL‐7 has been implicated in eosinophilic inflammatory responses in asthma through promotion of eosinophil activation and survival.^41^

Beyond these, MMP1 was significantly elevated only under the first BDR definition (10% FEV_1_). MMP‐1, an interstitial collagenase induced partly by IL‐4,^42^ contributes to bronchial hyperresponsiveness and asthma exacerbations through its role in airway remodeling.^43^ IL‐10 was significantly elevated only using the second BDR definition (FEV_1_ *z*‐score). Although traditionally regarded as anti‐inflammatory, IL‐10 has also been linked to T2 cytokine production, airway hyperreactivity, and virus‐induced exacerbations.^44^, ^45^, ^46^ The elevated IL‐10 levels in this study may reflect both ongoing disease activity and the known effect of corticosteroid therapy increasing endogenous IL‐10.^44^ These evidence might suggest that these inflammatory mediators might be involved in T2 and non‐T2 pathways. The absence of statistical significance under the 10% FEV_1_ definition may relate to differences in participant classification between BDR definitions, as well as the relatively larger sample size of the high‐BDR group identified under the *z*‐score definition, which may have slightly improved statistical power to detect differences in this cytokine.

Our study advances prior work from our group (Alizadeh Bahmani et al.), which found blood eosinophilia, defined by >300 cells/μL or >500 cells/μL, associated with higher IL‐5 levels.^6^ In our study, high BDR was not linked to blood eosinophils or IL‐5 but rather with elevations in IL‐4 and IL‐13, pointing to a distinct T2 signature. IL‐4 and IL‐13 signal through a shared receptor complex, making this pathway crucial in the pathophysiology of asthma and targeted for treatment.^31^ This emphasizes that BDR may provide additional insights into pediatric asthma endotypes. While blood eosinophils and IL‐5 remain critical markers, especially for identifying candidates for anti‐IL‐5 biologics, they are only part of the story. Blood eosinophil counts fluctuate widely, sometimes varying by over 40%, reducing the reliability of single time‐point measures.^47^ They also do not always mirror airway‐specific inflammation.^8^, ^9^ FeNO, though non‐invasive, is greatly attenuated by ICS therapy, limiting its discriminative use in treated cohorts such as ours.^24^ Although sputum analysis remains the clinical standard for assessing airway inflammation, its routine use in children is limited by procedural complexity.^4^, ^5^, ^6^ In contrast, BDR testing is non‐invasive, accessible, and easily repeatable, offering a valuable tool in clinical settings where frequent monitoring is needed. The relationship between high BDR, disease burden, and systemic inflammation highlights its utility in precision asthma care. Notably, elevated IL‐4 and IL‐13 across both BDR definitions could support the use of BDR to identify children likely to benefit from biologics targeting the IL‐4/IL‐13 axis (e.g., dupilumab), which has demonstrated efficacy in improving lung function and reducing exacerbations in pediatric MSA.^48^

Although asthma is primarily an airway disease, systemic cytokine elevations may reflect ongoing airway inflammation and immune activation, particularly in children with persistent or severe disease. Elevated serum IL‐4 and IL‐13 levels in children with high BDR may therefore represent indirect systemic signatures of underlying T2 airway inflammation and airway hyperresponsiveness. Nevertheless, serum cytokine concentrations should be interpreted cautiously, as systemic inflammatory markers do not fully reflect local airway inflammatory processes.

Our study has several strengths, including a comprehensive, multi‐dimensional phenotyping approach that integrates clinical, physiological, and molecular data. We evaluated BDR using two recommended thresholds and observed consistent results, indicating that the choice of cut‐off did not affect the associations. This supports BDR as a robust and reproducible marker of pediatric asthma phenotypes. Additionally, we applied rigorous adjustment for potential confounders to strengthen the validity of our findings. Another key strength is the focus on serum cytokine profiling, through which we are the first to link BDR with the immunologic heterogeneity of pediatric MSA, providing valuable insights that complement traditional clinical and physiological markers.

However, some limitations should be noted. The cross‐sectional design precludes causal inference. The relatively small number of children with high‐BDR may have limited statistical power for subgroup analyses and reduced the precision of some effect estimates. In addition, serum cytokines may not fully reflect airway inflammation due to compartmental differences. Finally, the inclusion of children with uniformly high medication adherence may introduce selection bias and limit generalizability to broader real‐world pediatric asthma populations, where adherence is often more variable.

Future studies should validate these findings in larger pediatric cohorts and assess the longitudinal stability of BDR‐related phenotypes. Evaluating whether BDR‐related inflammatory signatures predict long‐term outcomes and treatment responses will be critical. One promising direction is the use of oscillometry, which non‐invasively measures both large (proximal) and small (distal) airway function by assessing airway resistance and reactance and requires minimal patient cooperation.^49^ Its adoption in clinical and research settings could greatly enhance the assessment of lung function and treatment response in pediatric asthma. Moreover, smart inhaler technologies, which track medication use in real‐time, offer a valuable opportunity to obtain objective adherence data.^50^ Their use could overcome the limitations of self‐reported adherence measures and provide a more accurate understanding of how medication use relates to BDR, asthma control, and inflammatory patterns.

In conclusion, our results show that high BDR is associated with poorer asthma control. In addition, high BDR significantly associates with a distinct systemic inflammatory profile in children with MSA. This suggests that BDR can be a useful non‐invasive marker for asthma phenotyping.

## AUTHOR CONTRIBUTIONS

N. K. A. M. performed the analysis and drafted the initial version of the manuscript. M.I.A.‐A., and A.H.M.Z. contributed to the design of the analysis plan. All authors contributed to the acquisition of data, interpretation of the analysis, revision, drafting, and ensuring the accuracy and integrity of the analysis. All authors provided final approval of the version to be published.

## FUNDING INFORMATION

The SysPharmPediA consortium has been supported by ZonMW [project number: 9003035001], Ministry of Higher Education, Science and Innovation of the Republic of Slovenia (MVZI) [contract number C330‐16‐500106]; the German Ministry of Education and Research (BMBF) [project number FKZ 031 L0088]; Instituto de Salud Carlos III (ISCIII) through Strategic Action for Health Research (AES) and European Community (EC) within the Active and Assisted Living (AAL) Program framework [award numbers AC15/00015 and AC15/00058] under the frame of the ERACoSysMed JTC‐1 Call.

## CONFLICT OF INTEREST STATEMENT

A.H. Neerincx (AHN) reported support for the present manuscript via a grant paid to the institution from ERANET Systems Medicine and ZonMW. Mario Gorenjak (MG) disclosed reported support for the present manuscript including the SysPharmPedia grant co‐financed by the Ministry of Education, Science and Sport Slovenia (MIZS), and research core funding from the Slovenian Research Agency. Uroš Potočnik (UP) also reported support for the present manuscript from the SysPharmPedia grant, co‐financed by the Ministry of Higher Education, Science and Innovation Slovenia (MVZI), as well as core funding and a research grant from the Slovenian Research Agency. Maria Pino‐Yanes (MPY) reported support for the present manuscript from the Instituto de Salud Carlos III (ISCIII) and the SysPharmPedia grant from ERACoSysMed; furthermore, within the past 36 months, MPY received grants from the Spanish Ministry of Science, Innovation, and Universities (including the Ramon y Cajal program), grant support outside the submitted work from GlaxoSmithKline, Spain and CSL Behring, and funding from the Fundación Canaria Instituto de Investigación Sanitaria de Canarias (FIISC), alongside lecture fees from AstraZeneca Spain outside the submitted work. Michael Kabesch (MK) reported support from German Ministry of Education and Research and the European Research Council for this manuscript, and within the past 36 months reported grants/contracts from the European Union, German Ministry of Education and Research, and Bavarian Ministry of Health, consulting fees from AstraZeneca, and honoraria for lectures from EAACI, Novartis, Nutricia, and Pari. Anke H Maitland‐van der Zee (AHMZ) disclosed receiving grants (past 36 months) as the PI of the P4O2 consortium sponsored by Health Holland, involving private partners (AbbVie, Boehringer Ingelheim, Breathomix, Fluidda, Ortec Logiqcare, PeXA, Philips, Quantib‐U, Smartfish, Clear, SODAQ, Thirona, Roche, TopMD, Novartis, RespiQ), an unrestricted research grant from Boehringer Ingelheim, and a Vertex Innovation Award grant; her institution received consulting honoraria from Boehringer Ingelheim and Astra Zeneca; and she serves as the Chair of a DSMB of a study on BPD in neonates. Jan Willem Duitman (JWD) reported grants or contracts (past 36 months) from Abbvie, Boehringer Ingelheim, Dutch Lung Foundation, NWO, and Health~Holland. Susanne Vijverberg (SV) acts as the 3TR ABC Project Manager (past 36 months) for a project funded by the Innovative Medicines Initiative 2 Joint Undertaking (JU), supported by the European Union and EFPIA, listing pharmaceutical funders AstraZeneca, Sanofi & GSK, and holds a leadership role as an ERS Officer in Assembly 7 (Pediatrics). Olaia Sardón Prado (OSP) received support paid to her institution (past 36 months) from Faes Farma and Sanofi for attending the SENP 2025 congress. Finally, Susanne Harner (SHar) reported receiving payment or honoraria for lectures from Nutricia and Allergopharma, and support for travel from Nutricia (past 36 months). All other co‐authors, including Rene Lutter (RL), A.D. Kraneveld (ADK), Antoaneta A. Toncheva (AAT), Barbara S. Dierdorp (BSD), Christine Wolff (CW), E.G. Haarman (EGH), Leyre López‐Fernández (LLF), Mahmoud I. Abdel‐Aziz (MIAA), Nariman Kotb Abbas Metwally (NKAM), Paula Corcuera Elosegui (PCE), Dr. S.W.J. Terheggen‐Lagro (SWJTL), Simone Hashimoto (SH), Susanne Brandstetter (SB), and Tamara Dekker (TD) have no conflicts of interest to disclose.

## Supporting information


**Table S1:** Inflammatory markers measured by Luminex assay.
**Figure S1:** Assessment of Bronchodilator Response (BDR) Using Spirometry. This figure illustrates the spirometry procedure, where a patient performs inhalation and exhalation to measure lung function. The upper section represents the pre‐bronchodilator state, showing airway constriction and reduced airflow. The lower section represents the post‐bronchodilator state, where bronchodilator administration leads to airway relaxation and improved airflow. The graphs highlight Forced Expiratory Volume in 1 s (FEV_1_), a key parameter that measures the air breathed out forcefully within the first second after a deep breath. A significant improvement in FEV_1_ post‐bronchodilator is often used to confirm conditions like asthma.
**Figure S2:** Directed Acyclic Graph (DAG) illustrating the potential confounders considered in the analysis of the relationship between bronchodilator response (BDR), asthma control, and serum cytokines/chemokines. The DAG identifies key variables, including age, sex, ethnicity, BMI *z*‐score, baseline lung function, country, season of inclusion, GINA step, and current smoking exposure, guiding appropriate adjustment in statistical models to minimize confounding bias.
**Figure S3:** Overlap of high BDR classifications according to the two BDR definitions (>10% predicted and *z*‐score >0.78). All children identified by the >10% definition were also included in the *z*‐score group, with 8 additional children classified only by the *z*‐score.
**Table S2:** (A) Demographic and clinical characteristics of children with high and low BDR according to the FEV_1_ *z*‐score definition. (B) Lung function, white blood cell counts, and atopic sensitization in children with high and low BDR according to the FEV_1_ *z*‐score definition. (C) Asthma medication use and treatment steps in children with high and low BDR according to the FEV_1_ *z*‐score definition.
**Table S3:** Multi‐variate logistic regression model showing the association between BDR according to the FEV_1_ *z*‐score definition and asthma control after adjusting for confounders.
**Table S4:** Multi‐variate logistic regression model showing the association between BDR according to the 10% FEV_1_ definition and asthma control after adjusting for different confounders.
**Table S5:** Multi‐variate logistic regression model showing the association between BDR according to the FEV_1_ *z*‐score definition and asthma control after adjusting for different confounders.
**Figure S4:** Boxplots of serum cytokine and protein expression in children with high BDR and low BDR, shown separately for BDR definition: FEV_1_ *z*‐score >0.78 (high BDR). Boxplots display the distribution of significantly different cytokines and proteins between groups. Blue boxes represent the low BDR group, while red boxes represent the high BDR group. BDR, bronchodilator response; FEV_1_, forced expiratory volume in 1 s; CRP, C‐reactive protein, IL, interleukin; MCP, monocyte chemoattractant protein; MMP, matrix metalloproteinase; TARC, thymus and activation regulated chemokine; TNF‐α, tumor necrosis factor alpha.
**Figure S5:** Heatmaps of serum cytokine and chemokines profiles in children with high and low BDR shown for FEV_1_ *z*‐score >0.78 (high BDR). Each row represents a cytokine or protein, and each column represents an individual participant. Color intensity reflects relative expression levels, illustrating the separation between high BDR and low BDR groups; CRP, C‐reactive protein; IL, interleukin; MCP, monocyte chemoattractant protein; MMP, matrix metalloproteinase; TARC, thymus and activation regulated chemokine; TNF‐*α*, tumor necrosis factor alpha.
**Figure S6:** Adjusted associations between high bronchodilator response (BDR) and serum proteins levels. Associations for high BDR defined as FEV_1_ *z*‐score >0.78. Beta coefficients (*β*) represent the direction and magnitude of association, with FDR‐adjusted *p*‐values indicating statistical significance. All models were adjusted for age, sex, ethnicity, BMI *z*‐score, GINA treatment step, baseline lung function, study site, season of inclusion, and current smoking exposure. FEV_1_, forced expiratory volume in 1 s, BDR, bronchodilator response, IL, interleukin; TNF‐α, tumor necrosis factor alpha; MMP, matrix metalloproteinase; FDR, false discovery rate. A pseudo‐log X scale was used only for visualization purpose to allow plotting cytokines in different pg/mL ranges in one plot.
**Figure S7:** Protein–protein interaction network of high bronchodilator response (BDR)‐associated cytokines and inflammatory markers using the STRING database.
**Figure S8:** Pathway enrichment analysis of high BDR‐associated cytokines and proteins using Reactome via g:Profiler. Bar plot displays the top Reactome (REAC) pathways significantly enriched among the high BDR‐associated molecules (MMP‐1, IL‐4, IL‐13, IL‐6, IL‐7, IL‐8, IL‐10, TNF, and IL‐1β). The *y*‐axis shows the –log_1_₀(*p*adj) values for each pathway, with higher values indicating greater statistical significance. Adjusted *p*‐values (*p*adj) were calculated to account for multiple testing using the default false discovery rate (FDR) correction method implemented in g:Profiler. REAC, Reactome pathway database, *p*adj, adjusted *p*‐value, MMP, matrix metalloproteinase, IL, interleukin; TNF, tumor necrosis factor.

## References

[1] Pijnenburg MW , Fleming L . Advances in understanding and reducing the burden of severe asthma in children. Lancet Respir Med. 2020;8(10):1032‐1044. 32910897 10.1016/S2213-2600(20)30399-4

[2] Vijverberg SJH , Farzan N , Slob EMA , Neerincx AH , Maitland‐van der Zee AH . Treatment response heterogeneity in asthma: the role of genetic variation. Expert Rev Respir Med. 2018;12(1):55‐65. 29115880 10.1080/17476348.2018.1403318

[3] Ricciardolo FLM , Guida G , Bertolini F , Di Stefano A , Carriero V . Phenotype overlap in the natural history of asthma. Eur Respir Rev. 2023;32(168):220201. 37197769 10.1183/16000617.0201-2022 PMC10189644

[4] Dragonieri S , Bikov A , Capuano A , Scarlata S , Carpagnano GE . Methodological aspects of induced sputum. Adv Respir Med. 2023;91(5):397‐406. 37887074 10.3390/arm91050031 PMC10603896

[5] Djukanović R , Sterk PJ , Fahy JV , Hargreave FE . Standardised methodology of sputum induction and processing. Eur Respir J Suppl. 2002;37:1s‐2s. 12361359 10.1183/09031936.02.00000102

[6] Alizadeh Bahmani AH , Vijverberg SJH , Hashimoto S , et al. Association of blood inflammatory phenotypes and asthma burden in children with moderate‐to‐severe asthma. ERJ Open Res. 2024;10(6):222‐2024. 10.1183/23120541.00222-2024 PMC11647938 39687398

[7] Corneanu LE , Petriș OR , Lionte C , et al. Peripheral venipuncture in pediatric patients: a mini‐review of clinical practice and technological advances. J Clin Med. 2025;14(18):6397. 41010601 10.3390/jcm14186397 PMC12471072

[8] Hirano T , Matsunaga K . Measurement of blood eosinophils in asthma and chronic obstructive pulmonary disease. Intern Med. 2023;62(1):21‐25. 35431305 10.2169/internalmedicine.9339-22 PMC9876705

[9] Ullmann N , Bossley CJ , Fleming L , Silvestri M , Bush A , Saglani S . Blood eosinophil counts rarely reflect airway eosinophilia in children with severe asthma. Allergy. 2013;68(3):402‐406. 23347007 10.1111/all.12101

[10] Menzies‐Gow A , Mansur AH , Brightling CE . Clinical utility of fractional exhaled nitric oxide in severe asthma management. Eur Respir J. 2020;55(3):1901633. 31949116 10.1183/13993003.01633-2019

[11] Dweik RA , Boggs PB , Erzurum SC , et al. An official ATS clinical practice guideline: interpretation of exhaled nitric oxide levels (FENO) for clinical applications. Am J Respir Crit Care Med. 2011;184(5):602‐615. 21885636 10.1164/rccm.9120-11ST PMC4408724

[12] Sim YS , Lee JH , Lee WY , et al. Spirometry and bronchodilator test. Tuberc Respir Dis (Seoul). 2017;80(2):105‐112. 28416951 10.4046/trd.2017.80.2.105 PMC5392482

[13] Coverstone AM , Bacharier LB , Wilson BS , et al. Clinical significance of the bronchodilator response in children with severe asthma. Pediatr Pulmonol. 2019;54(11):1694‐1703. 31424170 10.1002/ppul.24473 PMC7015037

[14] Heffler E , Crimi C , Campisi R , et al. Bronchodilator response as a marker of poor asthma control. Respir Med. 2016;112:45‐50. 26823211 10.1016/j.rmed.2016.01.012

[15] Abdel‐Aziz MI , Neerincx AH , Vijverberg SJH , et al. A system pharmacology multi‐omics approach toward uncontrolled pediatric asthma. J Pers Med. 2021;11(6):484. 34071272 10.3390/jpm11060484 PMC8227234

[16] Cohen JL , Mann DM , Wisnivesky JP , et al. Assessing the validity of self‐reported medication adherence among inner‐city asthmatic adults: the medication adherence report scale for asthma. Ann Allergy Asthma Immunol. 2009;103(4):325‐331. 19852197 10.1016/s1081-1206(10)60532-7

[17] ATS/ERS recommendations for standardized procedures for the online and offline measurement of exhaled lower respiratory nitric oxide and nasal nitric oxide, 2005. Am J Respir Crit Care Med. 2005;171(8):912‐930. 15817806 10.1164/rccm.200406-710ST

[18] Nathan RA , Sorkness CA , Kosinski M , et al. Development of the asthma control test: a survey for assessing asthma control. J Allergy Clin Immunol. 2004;113(1):59‐65. 14713908 10.1016/j.jaci.2003.09.008

[19] Stanojevic S , Kaminsky DA , Miller MR , et al. ERS/ATS technical standard on interpretive strategies for routine lung function tests. Eur Respir J. 2022;60(1):2101499. 34949706 10.1183/13993003.01499-2021

[20] Quanjer PH , Ruppel GL , Langhammer A , et al. Bronchodilator response in FVC is larger and more relevant than in FEV(1) in severe airflow obstruction. Chest. 2017;151(5):1088‐1098. 28040521 10.1016/j.chest.2016.12.017

[21] Abdel‐Aziz MI , Hashimoto S , Neerincx AH , et al. Metabotypes are linked to uncontrolled childhood asthma, gut microbiota, and systemic inflammation. J Allergy Clin Immunol. 2025;156:339‐351. 40280190 10.1016/j.jaci.2025.04.017

[22] Alizadeh Bahmani AH , Slob EMA , Bloemsma LD , et al. Medication use in uncontrolled pediatric asthma: results from the SysPharmPediA study. Eur J Pharm Sci. 2023;181:106360. 36526249 10.1016/j.ejps.2022.106360

[23] Galant SP , Morphew T , Newcomb RL , Hioe K , Guijon O , Liao O . The relationship of the bronchodilator response phenotype to poor asthma control in children with normal spirometry. J Pediatr. 2011;158(6):953‐9. 21232757 10.1016/j.jpeds.2010.11.029 PMC3160763

[24] Matsunaga K , Hirano T , Akamatsu K , Minakata Y . Predictors for identifying the efficacy of systemic steroids on sustained exhaled nitric oxide elevation in severe asthma. Allergol Int. 2013;62(3):359‐365. 23880612 10.2332/allergolint.12-OA-0530

[25] Ferrer Galván M , Javier Alvarez Gutiérrez F , Romero Falcón A , Romero Romero B , Sáez A , Medina Gallardo JF . Is the bronchodilator test an useful tool to measure asthma control? Respir Med. 2017;126:26‐31. 28427546 10.1016/j.rmed.2017.03.008

[26] Fernandes AL , Amorim MM , Caetano LB , et al. Bronchodilator response as a hallmark of uncontrolled asthma: a randomised clinical trial. J Asthma. 2014;51(4):405‐410. 24404797 10.3109/02770903.2013.878845

[27] Kaminsky DA , He J , Henderson R , et al. Bronchodilator response does not associate with asthma control or symptom burden among patients with poorly controlled asthma. Respir Med. 2023;218:107375. 37536444 10.1016/j.rmed.2023.107375

[28] Madulara GM , Andaya AG . Effects of aeroallergen sensitization on symptom severity, pulmonary function, and bronchodilator response in children with bronchial asthma. Journal of Medicine, University of Santo Tomas. 2022;6(2):959‐970.

[29] Lombardi C , Savi E , Ridolo E , Passalacqua G , Canonica GW . Is allergic sensitization relevant in severe asthma? Which allergens may be culprit? World Allergy Organ J. 2017;10(1):2. 28101292 10.1186/s40413-016-0138-8 PMC5219672

[30] Forno E , Celedon JC . Asthma and ethnic minorities: socioeconomic status and beyond. Curr Opin Allergy Clin Immunol. 2009;9(2):154‐160. 19326508 10.1097/aci.0b013e3283292207 PMC3920741

[31] Pelaia C , Heffler E , Crimi C , et al. Interleukins 4 and 13 in asthma: key pathophysiologic cytokines and Druggable molecular targets. Front Pharmacol. 2022;13:851940. 35350765 10.3389/fphar.2022.851940 PMC8957960

[32] Adrish M , Akuthota P . Approach to non‐type 2 asthma. Respir Med. 2023;216:107327. 37307904 10.1016/j.rmed.2023.107327

[33] Sze E , Bhalla A , Nair P . Mechanisms and therapeutic strategies for non‐T2 asthma. Allergy. 2020;75(2):311‐325. 31309578 10.1111/all.13985

[34] Padrón‐Morales J , García‐Solaesa V , Isidoro‐García M , et al. Implications of cytokine genes in allergic asthma. Allergol Immunopathol (Madr). 2014;42(6):603‐608. 24731768 10.1016/j.aller.2013.11.006

[35] Barnes PJ . The cytokine network in asthma and chronic obstructive pulmonary disease. J Clin Invest. 2008;118(11):3546‐3556. 18982161 10.1172/JCI36130 PMC2575722

[36] Caucheteux SM , Hu‐Li J , Guo L , et al. IL‐1β enhances inflammatory TH2 differentiation. J Allergy Clin Immunol. 2016;138(3):898‐901. 27212084 10.1016/j.jaci.2016.02.033 PMC5014664

[37] Choi JP , Kim YS , Kim OY , et al. TNF‐alpha is a key mediator in the development of Th2 cell response to inhaled allergens induced by a viral PAMP double‐stranded RNA. Allergy. 2012;67(9):1138‐1148. 22765163 10.1111/j.1398-9995.2012.02871.x

[38] Neveu WA , Allard JL , Raymond DM , et al. Elevation of IL‐6 in the allergic asthmatic airway is independent of inflammation but associates with loss of central airway function. Respir Res. 2010;11(1):28. 20205953 10.1186/1465-9921-11-28 PMC2842243

[39] Jackson DJ , Bacharier LB , Calatroni A , et al. Serum IL‐6: a biomarker in childhood asthma? J Allergy Clin Immunol. 2020;145(6):1701‐4. 32004524 10.1016/j.jaci.2020.01.021 PMC7282967

[40] Chu DK , Al‐Garawi A , Llop‐Guevara A , et al. Therapeutic potential of anti‐IL‐6 therapies for granulocytic airway inflammation in asthma. Allergy Asthma Clin Immunol. 2015;11(1):14. 25878673 10.1186/s13223-015-0081-1 PMC4397814

[41] Kelly EA , Koziol‐White CJ , Clay KJ , et al. Potential contribution of IL‐7 to allergen‐induced eosinophilic airway inflammation in asthma. J Immunol. 2009;182(3):1404‐1410. 19155487 10.4049/jimmunol.182.3.1404 PMC2851244

[42] Sasaguri T , Arima N , Tanimoto A , Shimajiri S , Hamada T , Sasaguri Y . A role for interleukin 4 in production of matrix metalloproteinase 1 by human aortic smooth muscle cells. Atherosclerosis. 1998;138(2):247‐253. 9690907 10.1016/s0021-9150(97)00296-7

[43] Naveed SU , Clements D , Jackson DJ , et al. Matrix Metalloproteinase‐1 activation contributes to airway smooth muscle growth and asthma severity. Am J Respir Crit Care Med. 2017;195(8):1000‐1009. 27967204 10.1164/rccm.201604-0822OC PMC5422648

[44] Ogawa Y , Duru EA , Ameredes BT . Role of IL‐10 in the resolution of airway inflammation. Curr Mol Med. 2008;8(5):437‐445. 18691071 10.2174/156652408785160907 PMC9159958

[45] Qian G , Jiang W , Sun D , et al. B‐cell‐derived IL‐10 promotes allergic sensitization in asthma regulated by Bcl‐3. Cell Mol Immunol. 2023;20(11):1313‐1327. 37653127 10.1038/s41423-023-01079-w PMC10616210

[46] Grissell TV , Powell H , Shafren DR , et al. Interleukin‐10 gene expression in acute virus‐induced asthma. Am J Respir Crit Care Med. 2005;172(4):433‐439. 15894599 10.1164/rccm.200412-1621OC

[47] Mathur SK , Fichtinger PS , Evans MD , Schwantes EA , Jarjour NN . Variability of blood eosinophil count as an asthma biomarker. Ann Allergy Asthma Immunol. 2016;117(5):551‐553. 27590639 10.1016/j.anai.2016.08.010 PMC5085834

[48] Bacharier LB , Maspero JF , Katelaris CH , et al. Dupilumab in children with uncontrolled moderate‐to‐severe asthma. N Engl J Med. 2021;385(24):2230‐2240. 34879449 10.1056/NEJMoa2106567

[49] Smith EF , Bradshaw TK , Urs RC , et al. Oscillometry and spirometry are not interchangeable when assessing the bronchodilator response in children and young adults born preterm. Pediatr Pulmonol. 2023;58(11):3122‐3132. 37539845 10.1002/ppul.26632 PMC10947568

[50] Reddel HK , Bateman ED , Schatz M , Krishnan JA , Cloutier MM . A practical guide to implementing SMART in asthma management. J Allergy Clin Immunol Pract. 2022;10(1s):S31. 34666208 10.1016/j.jaip.2021.10.011

