# Supplementary material for: Bronchodilator response is linked with uncontrolled moderate‐to‐severe childhood asthma and elevated IL‐4 and IL‐13
Source: Pediatr Allergy Immunol. 2026 Jun 8;37(6):e70392. doi: 10.1111/pai.70392 (PMC13244407; doi:10.1111/pai.70392)
Supplement: Supplementary file 1 — Table S1: Inflammatory markers measured by Luminex assay. Figure S1: Assessment of Bronchodilator Response (BDR) Using Spirometry. This figure illustrates the spirometry procedure, where a patient performs inhalation and exhalation to measure lung function. The upper section represents the pre‐bronchodilator state, showing airway constriction and reduced airflow. The lower section represents the post‐bronchodilator state, where bronchodilator administration leads to airway relaxation and improved airflow. The graphs highlight Forced Expiratory Volume in 1 s (FEV1), a key parameter that measures the air breathed out forcefully within the first second after a deep breath. A significant improvement in FEV1 post‐bronchodilator is often used to confirm conditions like asthma. Figure S2: Directed Acyclic Graph (DAG) illustrating the potential confounders considered in the analysis of the relationship between bronchodilator response (BDR), asthma control, and serum cytokines/chemokines. The DAG identifies key variables, including age, sex, ethnicity, BMI z‐score, baseline lung function, country, season of inclusion, GINA step, and current smoking exposure, guiding appropriate adjustment in statistical models to minimize confounding bias. Figure S3: Overlap of high BDR classifications according to the two BDR definitions (>10% predicted and z‐score >0.78). All children identified by the >10% definition were also included in the z‐score group, with 8 additional children classified only by the z‐score. Table S2: (A) Demographic and clinical characteristics of children with high and low BDR according to the FEV1 z‐score definition. (B) Lung function, white blood cell counts, and atopic sensitization in children with high and low BDR according to the FEV1 z‐score definition. (C) Asthma medication use and treatment steps in children with high and low BDR according to the FEV1 z‐score definition. Table S3: Multi‐variate logistic regression model showing the association between BD [file PAI-37-e70392-s001.zip › Supplementary_material_PAI_04-06-2026.docx]

Online Supplementary Material

**Bronchodilator response is linked with uncontrolled moderate-to-severe childhood asthma and elevated IL-4 and IL-13**

Nariman K A Metwally^1,2,3,4,5^, Simone Hashimoto^1,2,3,4^, Susanne J H Vijverberg^1,2,3^, Anne H Neerincx^1^, Barbara S Dierdorp^2,6^, Tamara Dekker^2,6^, Eric G Haarman^4^, Jan Willem Duitman^1,2,6^, Mario Gorenjak^7^, Antoaneta A Toncheva^8^, Susanne Harner^8^, Susanne Brandstetter^9^, Christine Wolff^9^, Paula Corcuera-Elosegui^10^, Leyre López-Fernández^10^, Olaia Sardón-Prado^10,11^, Maria Pino-Yanes^12,13,14^, Uroš Potočnik^7,15,16^, Michael Kabesch^8,9^, Aletta D Kraneveld^17^, René Lutter^1,2,6^, Suzanne W J Terheggen-Lagro^4^, Mahmoud I Abdel-Aziz^1,2,3,18,19^#, Anke H Maitland-van der Zee^1,2,3,4,18^#; on behalf of the SysPharmPediA Consortium

1 Department of Pulmonary Medicine, Amsterdam UMC, University of Amsterdam, Amsterdam, the Netherlands.

2 Amsterdam Institute for Infection and Immunity, Inflammatory diseases, Amsterdam, the Netherlands.

3 Amsterdam Public Health, Personalized Medicine, Amsterdam, The Netherlands.

4 Department of Pediatric Pulmonology and Allergy, Emma Children’s Hospital, Amsterdam UMC, Amsterdam, The Netherlands.

5 Department of Public and Occupational Health, Amsterdam UMC, University of Amsterdam, Amsterdam, the Netherlands.

6 Department of Experimental Immunology, Amsterdam UMC, University of Amsterdam, Amsterdam, the Netherlands.

7 Center for Human Molecular Genetics and Pharmacogenomics, Faculty of Medicine, University of Maribor, Maribor, Slovenia.

8 Department of Pediatric Pneumology and Allergy, University Children’s Hospital Regensburg (KUNO), Regensburg, Germany.

9 University Children's Hospital, University of Regensburg, Regensburg, Germany.

10 Division of Pediatric Respiratory Medicine, Donostia University Hospital, San Sebastián, Spain.

11 Department of Pediatrics. Faculty of Medicine and Nursing. University of the Basque Country (UPV/EHU), San Sebastián, Spain.

12 Genomics and Health Group, Department of Biochemistry, Microbiology, Cell Biology, and Genetics, Universidad de La Laguna (ULL), La Laguna, Tenerife, Spain.

13 CIBER de Enfermedades Respiratorias, Instituto de Salud Carlos III, Madrid, Spain.

14 Instituto de Tecnologías Biomédicas (ITB), Universidad de La Laguna (ULL), La Laguna, Tenerife, Spain.

15 Faculty of Chemistry and Chemical Engineering, University of Maribor, Smetanova ulica 17,

2000 Maribor, Slovenia.

16 Department for Science and Research, University Medical Centre Maribor, Ljubljanska ulica 5,

2000 Maribor, Slovenia.

17 Division of Pharmacology, Utrecht Institute for Pharmaceutical Sciences, Faculty of Science, Utrecht University, 3584 CG Utrecht, The Netherlands.

18 Department of Genetics, UMC Groningen, University of Groningen, Groningen, Netherlands .

19 Department of Clinical Pharmacy, Faculty of Pharmacy, Assiut University, Assiut, Egypt.

# Contributed equally

## **Supplementary methods**

## **Ethical considerations**

The study was conducted in accordance with the Declaration of Helsinki and was approved by the local Medical ethics committee of all participating study centers before participant enrollment (ethics committee of University Regensburg, Germany (18-1034-101); Clinical Research Ethics Committee of the Basque Country, Spain (PI2015075 (SO)); Medical Ethics Committee of the University Medical Center Utrecht (UMC Utrecht), Utrecht, the Netherlands (NL55788.041.15); National Medical Ethics Committee, Slovenia (0120-569/2017/4)). Written informed consent was obtained from all participants or their parents/caregivers, with additional consent from the children when appropriate.

## **Measurement of inflammatory markers**

### **Fractional Exhaled Nitric Oxide (FeNO)**

Measured to assess airway inflammation, using specific equipment across the study centers at the recommended flow rate of 50 mL/s based on the ERS/ATS guidelines (1).

### **Atopic sensitization**

Information on Evidence of atopy was obtained from patients’ medical histories. Patients were classified as atopic when showing sensitization at any time to one or more allergens, as evidenced by a positive skin prick test (SPT, wheal diameter ≥3 mm) and/or an elevated level of allergen-specific serum IgE (≥0.35 kU/L).

### **Blood samples collection and preparation**

Peripheral venous blood was collected from each participant during the baseline study visit via venipuncture. EDTA blood was used for differential blood count and stored at room temperature, analyzed within two hours. Blood tubes were centrifuged, and serum was extracted, divided into aliquots, and stored at -80°C until analysis.

### **White blood inflammatory cell count**

Differential white blood inflammatory cell count (such as eosinophils, and neutrophils) were measured in fresh peripheral blood on the same day of the baseline visits using fluorescence flow cytometry as described (2).

**Table S1: Inflammatory markers measured by Luminex assay**

| Protein Full Name | Abbreviation |
| --- | --- |
| C-reactive protein | CRP |
| Cluster of Differentiation 14 | CD14 |
| Pulmonary and Activation-Regulated Chemokine | PARC |
| Matrix Metalloproteinase 9 | MMP-9 |
| Growth-Regulated Alpha Protein | GRO-α |
| Interleukin 17 | IL-17 |
| Vascular Endothelial Growth Factor | VEGF |
| Interleukin 1 alpha | IL-1α |
| Thymic Stromal Lymphopoietin | TSLP |
| Monocyte Chemoattractant Protein 4 | MCP-4 |
| Monocyte Chemoattractant Protein 1 | MCP-1 |
| Macrophage Inflammatory Protein 3 beta | MIP-3β |
| Interferon Gamma-Induced Protein 10 | IP-10 |
| Interleukin 1 beta | IL-1β |
| Interleukin 6 | IL-6 |
| Interleukin 8 | IL-8 |
| Interleukin 13 | IL-13 |
| Interleukin 23 | IL-23 |
| Macrophage Colony-Stimulating Factor | M-CSF |
| Matrix Metalloproteinase 3 | MMP-3 |
| Osteoblast-Specific Factor 2 | OSF-2 |
| Tumor Necrosis Factor alpha | TNF-α |
| Thymus and Activation-Regulated Chemokine | TARC |
| Macrophage-Derived Chemokine | MDC |
| Granulocyte-Macrophage Colony-Stimulating Factor | GM-CSF |
| Interleukin 4 | IL-4 |
| Interleukin 7 | IL-7 |
| Interleukin 10 | IL-10 |
| Interleukin 18 | IL-18 |
| Interleukin 33 | IL-33 |
| Matrix Metalloproteinase 1 | MMP-1 |
| Matrix Metalloproteinase 12 | MMP-12 |
| Receptor for Advanced Glycation End Products | RAGE |
| Interleukin 5 | IL-5 |
| Tissue Inhibitor of Metalloproteinases 1 | TIMP-1 |
| Tissue Inhibitor of Metalloproteinases 2 | TIMP-2 |
| Tissue Inhibitor of Metalloproteinases 3 | TIMP-3 |
| Tissue Inhibitor of Metalloproteinases 4 | TIMP-4 |
| Interleukin 9 | IL-9 |
| Interleukin 22 | IL-22 |


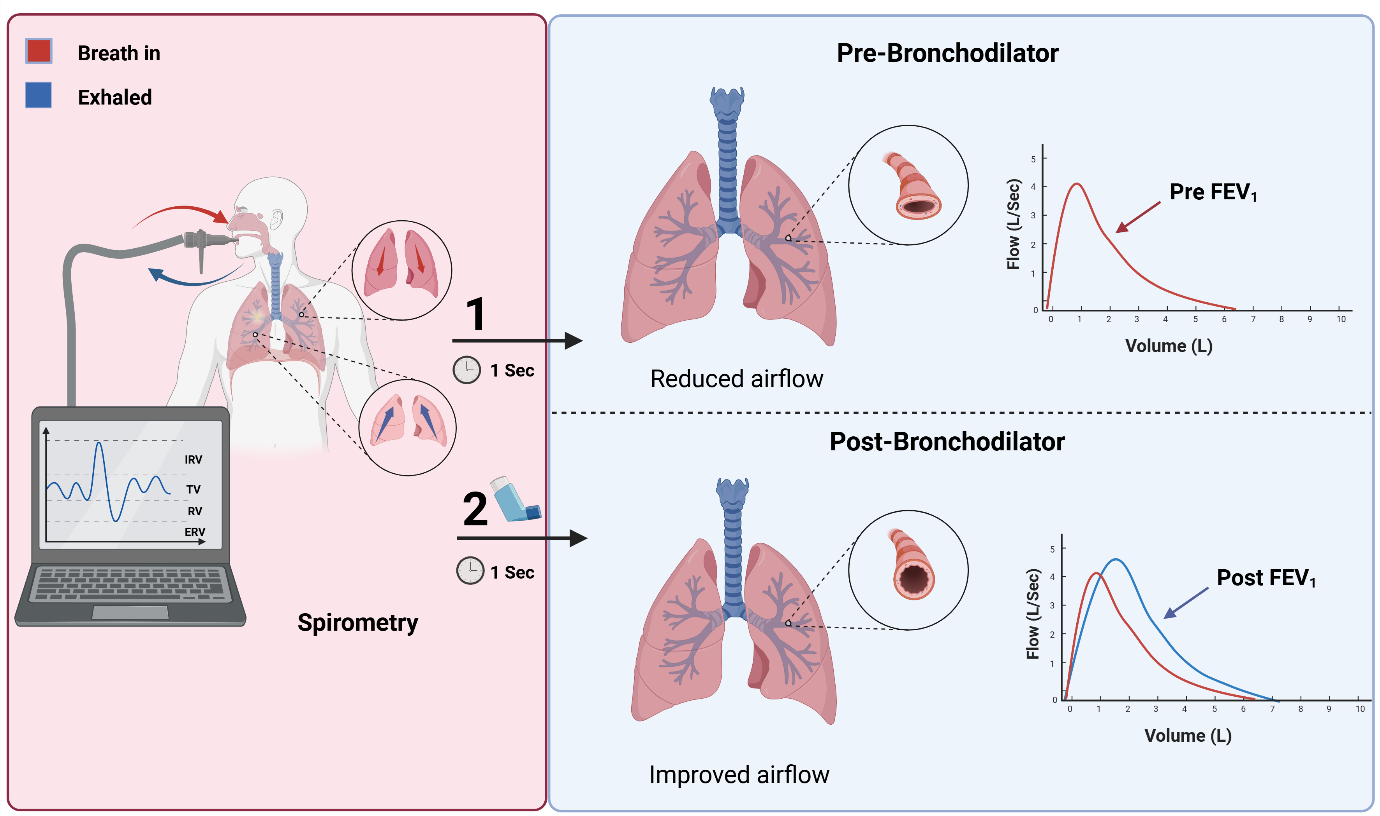


Figure S1: Assessment of Bronchodilator Response (BDR) Using Spirometry. This figure illustrates the spirometry procedure, where a patient performs inhalation and exhalation to measure lung function. The upper section represents the pre-bronchodilator state, showing airway constriction and reduced airflow. The lower section represents the post-bronchodilator state, where bronchodilator administration leads to airway relaxation and improved airflow. The graphs highlight Forced Expiratory Volume in 1 second (FEV₁), a key parameter that measures the air breathed out forcefully within the first second after a deep breath. A significant improvement in FEV₁ post-bronchodilator is often used to confirm conditions like asthma. Created in BioRender.


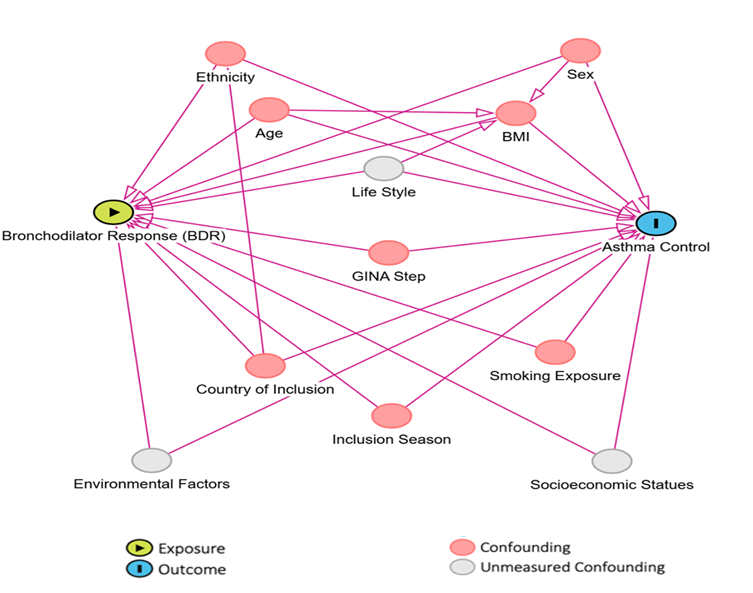


Figure S2: Directed Acyclic Graph (DAG) illustrating the potential confounders considered in the analysis of the relationship between bronchodilator response (BDR), asthma control, and serum cytokines/chemokines. The DAG identifies key variables, including age, sex, ethnicity, BMI z-score, baseline lung function, country, season of inclusion, GINA step, and current smoking exposure, guiding appropriate adjustment in statistical models to minimize confounding bias.

## **Supplementary Results**


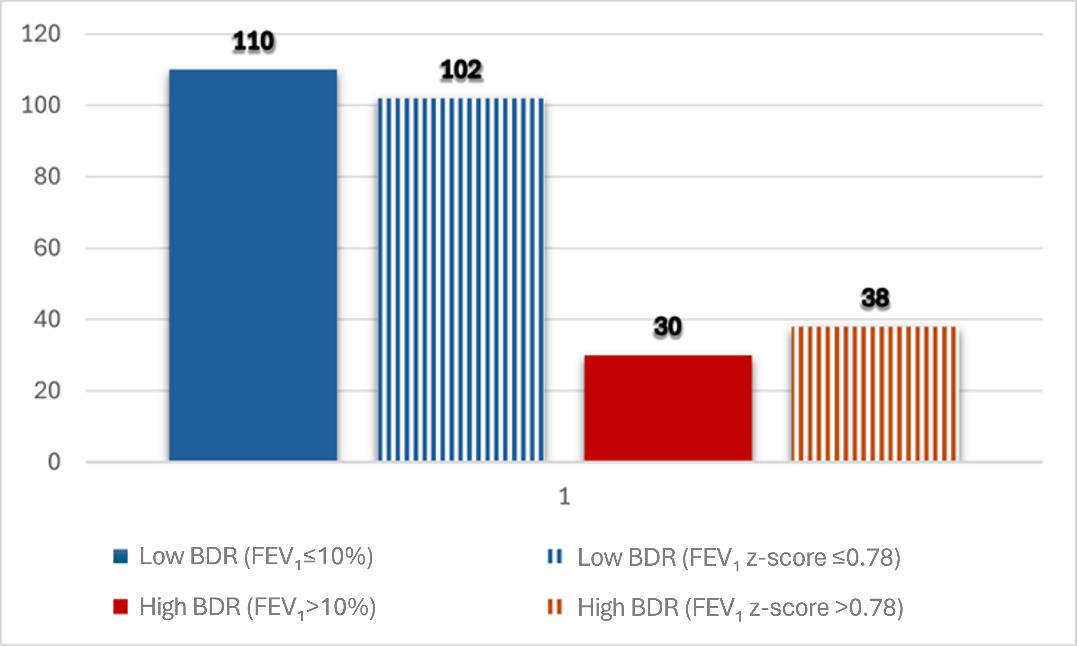


Figure S3: Overlap of high BDR classifications according to the two BDR definitions (>10% predicted and z-score >0.78). All children identified by the >10% definition were also included in the z-score group, with 8 additional children classified only by the z-score.

**Table S2:**

**Table S2 A. Demographic and clinical characteristics of children with high and low  BDR according to the** **FEV₁ z-score definition.**

| **Characteristics** | **Low BDR (ΔFEV₁ ≤0.78)**  **(n=102)** | **High BDR** **(ΔFEV₁ >0.78)**  **(n=38)** | **P value** |
| --- | --- | --- | --- |
| **Age in years, median (IQR)** | 11.59 (9.64, 13.96) | 12.95 (10.10, 14.00) | 0.536 |
| **Sex ( Female), n (%)** | 43/110 (39%) | 12/30 (40%) | 0.928 |
| **Ethnicity, n (%)** |  |  | **0.028** |
| - **Caucasian** | **81/101 (80%)** | **26/38 (68%)** |  |
| - **Latino** | 9/101 (9%) | 1/38 (3%) |  |
| - **African** | 4/101 (4%) | 2/38 (5%) |  |
| - **Asian** | 0/101 (0%) | 2/38 (5%) |  |
| - **Mixed/others** | 7/101 (7%) | 7/38 (18%) |  |
| **BMI z-score, median (IQR)** | 0.63 (-0.28, 1.38) | 0.47 (-0.48, 1.47) | 0.809 |
| **Birth and Early Life Factors, n (%)** |  |  |  |
| - **Mode of delivery, (Caesarean section)** | 20/99 (20%) | 6/37 (16%) | 0.599 |
| - **Breast feeding > 4 months** | 4.00 (0.00, 9.00)  (n=97) | 4.00 (1.00, 11.00)  (n=37) | 0.661 |
| **Smoking Exposure , n (%)** |  |  |  |
| - **Smoking exposure during pregnancy** | 25/95 (26%) | 9/32 (28%) | 0.842 |
| - **Current smoke exposure** | 29/100 (29%) | 11/35 (31%) | 0.787 |
| **Country of Inclusion, n (%)** |  |  | **0.015** |
| - **Spain** | 42/102 (41%) | 8/38 (21%) |  |
| - **Germany** | 29/102 (28%) | 8/38 (21%) |  |
| - **The Netherlands** | 17/102 (17%) | 15/38 (39%) |  |
| - **Slovenia** | 14/102 (14%) | 7/38 (18%) |  |
| **Inclusion season, n (%)** |  |  | **0.042** |
| - **Winter** | 23/102 (23%) | 4/38 (11%) |  |
| - **Spring** | 31/102 (30%) | 7/38 (18%) |  |
| - **Summer** | 27/102 (26%) | 19/38 (50%) |  |
| - **Autumn** | 21/102 (21%) | 8/38 (21%) |  |
| **Clinical Status, n (%)** |  |  |  |
| - **Uncontrolled Asthma †** | 59/102 (58%) | 29/38 (76%) | **0.044** |
| - **(childhood) Asthma Control Test ((c) ACT) z-score, median (IQR)** | 1.03 (0.30, 1.55)  (n=101) | 0.55 (0.12, 1.28)  (n=37) | 0.065 |
| - **Severe asthma exacerbations in the past year** | 47/102 (46%) | 25/38 (66%) | **0.038** |

**Table S2 B. Lung function, white blood cell counts, and atopic sensitization in children with high and low BDR according to the FEV₁ z-score definition**

| **Characteristics** | **Low BDR (ΔFEV₁ ≤0.78)**  **(n=102)** | **High BDR (ΔFEV₁ >0.78)**  **(n=38)** | **P value** |
| --- | --- | --- | --- |
| **Lung Function** |  |  |  |
| - **FEV₁ pre-salbutamol % predicted, median (IQR)** | 96.73 (86.69, 105.14) | 85.14 (78.76, 96.03) | **<0.001** |
| - **FEV₁ post-salbutamol % predicted, median (IQR)** | 99.66 (89.60, 108.16) | 99.08 (91.99, 109.13) | 0.923 |
| - **FEV₁ pre-salbutamol z-score, median (IQR)** | -0.29 (-1.15, 0.44) | -1.25 (-1.80, -0.34) | **<0.001** |
| - **FEV₁ post-salbutamol z-score, median (IQR)** | -0.03 (-0.88, 0.72) | -0.08 (-0.68, 0.77) | 0.929 |
| - **FeNO (ppb), median (IQR)** | 12.95 (8.15, 33.28)  (n=90) | 20.70 (10.38, 46.83)  (n=32) | 0.173 |
| **White blood cell count (×10^9^/L), median (IQR)** |  |  |  |
| - **Eosinophil** | 0.38 (0.20, 0.65)  (n=89) | 0.34 (0.23, 0.60)  (n=34) | 0.989 |
| - **Neutrophil** | 3.10 (2.43, 4.00)  (n=89) | 3.77 (2.43, 4.80)  (n=34) | 0.118 |
| - **Lymphocyte** | 2.56 (2.23, 3.10)  (n=89) | 2.76 (2.17, 3.10)  (n=34) | 0.83 |
| - **Eosinophilia, n (%):** |  |  |  |
| - **> 0.3×10^9^/L** | 57/89 (64%) | 21/34 (62%) | 0.814 |
| - **> 0.5×10^9^/L** | 33/89 (37%) | 11/34 (32%) | 0.625 |
| **Atopy and Allergic Sensitization, n (%) ‡** |  |  |  |
| - **Atopy** | 86/98 (88%) | 33/36 (92%) | 0.759 |
| - **Aeroallergen combined** | 86/99 (87%) | 33/37 (89%) | 1 |
| - **HDM** | 70/96 (73%) | 25/37 (68%) | 0.541 |
| - **Grass pollen** | 52/97 (54%) | 23/36 (64%) | 0.288 |
| - **Mold** | 8/81 (10%) | 5/29 (17%) | 0.322 |
| - **Cat** | 28/93 (30%) | 18/32 (56%) | **0.008** |
| - **Dog** | 21/91 (23%) | 17/28 (61%) | **< 0.001** |
| - **IgE, abnormal** | 66/78 (85%) | 32/35 (91%) | 0.385 |
| - **Allergic rhinitis (ever), n (%)** | 76/96 (79%) | 25/37 (68%) | 0.161 |
| - **Atopic dermatitis Eczema** | 39/93 (42%) | 13/35 (37%) | 0.623 |

**Table S3 C. Asthma medication use and treatment steps in children with high and low BDR according to** **the FEV₁ z-score definition**

| **Characteristics** | **Low BDR (ΔFEV₁ ≤0.78)**  **(n=102)** | **High BDR (ΔFEV₁ >0.78)**  **(n=38)** | **P value** |
| --- | --- | --- | --- |
| **Asthma Medication n (%)** |  |  |  |
| - **ICS** | 102 (100%) | 38 (100%) |  |
| - **SABA** | 94/102 (92%) | 36/38 (95%) | 0.728 |
| - **LABA** | 98/102 (96%) | 34/38 (89%) | 0.213 |
| - **OCS** | 1/102 (1%) | 3/38 (8%) | 0.061 |
| - **LTRA** | 15/102 (15%) | 10/38 (26%) | 0.111 |
| - **Anticholinergics** | 11/102 (11%) | 6/38 (16%) | 0.4 |
| - **Biologics (Omalizumab/** - **Mepolizumab)** | 7/102 (7%) | 8/38 (21%) | **0.028** |
| **GINA Steps, n (%)** |  |  | 0.067 |
| - **Step 3 (GINA)** | 50/102 (49%) | 13/38 (34%) |  |
| - **Step 4 (GINA)** | 44/102 (43%) | 17/38 (45%) |  |
| - **Step 5 (GINA)** | 8/102 (8%) | 8/38 (21%) |  |
| **MARS-5 (>=21), n (%)** | 89/94 (95%) | 28/32 (88%) | 0.23 |

Footnote Table 1: Demographic, clinical, and treatment characteristics are presented for the total cohort and stratified by BDR status according to the FEV₁ z-score increase >0.78 definition (high BDR) and low BDR group are those who did not meet the respective BDR criterion. Continuous variables are presented as medians with interquartile ranges, while categorical variables are shown as counts and percentages. Comparisons between high BDR and low BDR groups were conducted using the Mann-Whitney U test for continuous data and either the chi-square test or Fisher’s exact test for categorical data, as appropriate. A p-value below 0.05 was showed evidence of a significant difference between groups. **†** Uncontrolled asthma is characterized by a (childhood) Asthma Control Test (cACT) score of 19 or below and/or the occurrence of severe exacerbations within the previous year that necessitated hospitalization, emergency room visits, or the use of oral corticosteroids (OCS). ‡ Atopic sensitization is defined based on a physician-documented history of sensitization to airborne allergens, demonstrated by a positive skin prick test (wheal diameter ≥3 mm) and/or elevated allergen-specific IgE levels (≥0.35 kU/L).

**Supplementary Table S3: Multi-variate logistic regression model showing the association between BDR according to the FEV₁ z-score definition and asthma control after adjusting for confounders.**

| **Characteristic (n=134)** | **OR** | **SE** | **95% CI** | **p-value** |
| --- | --- | --- | --- | --- |
| **BDR (FEV₁ z-score >0.78)** | **3.06** | 0.543 | 1.09, 9.39 | **0.039** |
| **Baseline FEV₁ (L)** | 1.14 | 0.512 | 0.42, 3.15 | 0.801 |
| **Age (years)** | 0.93 | 0.135 | 0.71, 1.21 | 0.581 |
| **Sex(Male)** | 0.79 | 0.451 | 0.32, 1.90 | 0.595 |
| **BMI z-score** | 1.01 | 0.178 | 0.71, 1.44 | 0.945 |
| **Ethnicity (Other/Mixed)** | 2.57 | 0.669 | 0.73, 10.7 | 0.159 |
| **Country of inclusion †** |  |  |  |  |
| - Netherlands | 1.44 | 0.749 | 0.33, 6.41 | 0.625 |
| - Slovenia | 1.39 | 0.709 | 0.34, 5.62 | 0.646 |
| - Spain | 2.38 | 0.584 | 0.76, 7.66 | 0.138 |
| **Inclusion season ‡** |  |  |  |  |
| - Spring | 0.85 | 0.581 | 0.27, 2.66 | 0.783 |
| - Summer | 0.53 | 0.655 | 0.14, 1.88 | 0.337 |
| - Winter | 1.19 | 0.677 | 0.32, 4.63 | 0.793 |
| **GINA treatments step §** |  |  |  |  |
| - Step4 | 2.59 | 0.496 | 0.99, 7.06 | **0.055** |
| - Step5 | 14.1 | 1.18 | 1.94, 297 | **0.025** |
| **Current smoking exposure** | 0.78 | 0.473 | 0.31, 1.97 | 0.595 |

Footnote Table S3: Multi-variate logistic regression model showing the association between high bronchodilator response (BDR) according to the FEV₁ z-score definition and asthma control after adjusting for different confounders. Odds ratios (ORs) and their 95% confidence intervals (CIs) for uncontrolled asthma are shown for each variable, with controlled asthma as the reference group. Variables include high bronchodilator response (BDR, defined as FEV₁ z-score increase >0.78), baseline FEV₁, age, sex, ethnicity, country of inclusion, BMI z-score, season of inclusion, GINA treatment step, and current smoking exposure. Abbreviations: Abbreviations: BDR = bronchodilator response; FEV₁= forced expiratory volume in one second; OR= odds ratio; CI= confidence interval; SE= standard error; BMI= body mass index; GINA= Global Initiative for Asthma. † Germany was selected randomly as a reference group for comparison of country of inclusion, ‡ Autumn was selected randomly as a reference group for comparison of Inclusion season, § Step 3 was selected randomly as a reference group for comparison of GINA Treatment Step

**Table S4: Multi-variate logistic regression model showing the association between BDR according to the 10% FEV₁ definition and asthma control after adjusting for different confounders**.

| **Characteristic** | **OR** | **SE** | **95% CI** | **p-value** |
| --- | --- | --- | --- | --- |
| **BDR (FEV₁ z-score >10)** | 3.20 | 0.580 | 1.09, 11.0 | **0.045** |
| **Baseline FEV₁ (L)** | 0.85 | 0.276 | 0.49, 1.47 | 0.566 |
| **BMI z-score** | 1.08 | 0.166 | 0.78, 1.50 | 0.645 |
| **Country of Inclusion †** |  |  |  |  |
| - Netherlands | 1.76 | 0.683 | 0.47, 6.93 | 0.407 |
| - Slovenia | 1.42 | 0.697 | 0.36, 5.64 | 0.614 |
| - Spain | 2.49 | 0.563 | 0.84, 7.70 | 0.105 |
| **Inclusion season ‡** |  |  |  |  |
| - Spring | 0.92 | 0.577 | 0.29, 2.86 | 0.887 |
| - Summer | 0.64 | 0.623 | 0.18, 2.15 | 0.479 |
| - Winter | 1.21 | 0.663 | 0.33, 4.54 | 0.778 |
| **GINA Treatment Step §** |  |  |  |  |
| - Step4 | 3.07 | 0.484 | 1.21, 8.18 | **0.021** |
| - Step5 | 19.0 | 1.14 | 2.88, 383 | **0.010** |
| **Current Smoking Exposure** | 0.83 | 0.449 | 0.34, 2.01 | 0.673 |

Footnote Table S4: Multi-variate logistic regression model showing the association between high bronchodilator response (BDR) according to the FEV₁ >10% definition and asthma control after adjusting for different confounders. Odds ratios (ORs) and 95% confidence intervals (CIs) for uncontrolled asthma are shown for each variable, with controlled asthma as the reference group. Variables include high bronchodilator response (BDR, defined as >10% increase in predicted FEV₁), baseline FEV₁, country of inclusion, BMI z-score, season of inclusion, GINA treatment step, and current smoking exposure. Abbreviations: BDR = bronchodilator response; FEV₁= forced expiratory volume in one second; OR= odds ratio; CI= confidence interval; SE= standard error; BMI= body mass index; GINA= Global Initiative for Asthma. † Germany was selected randomly as a reference group for comparison of country of inclusion, ‡ Autumn was selected randomly as a reference group for comparison of Inclusion season, § Step 3 was selected as a reference group for comparison of GINA Treatment Step.

**Table S5: Multi-variate logistic regression model showing the association between BDR according to the FEV₁ z-score definition and asthma control after adjusting for different confounders.**

| **Characteristic** | **OR** | **SE** | **95% CI** | **p-value** |
| --- | --- | --- | --- | --- |
| **BDR (FEV₁ z-score > 0.78)** | 2.88 | 0.529 | 1.06, 8.62 | **0.046** |
| **Baseline FEV₁ (L)** | 0.86 | 0.277 | 0.49, 1.48 | 0.581 |
| **BMI z-score** | 1.10 | 0.166 | 0.79, 1.53 | 0.564 |
| **Country of Inclusion †** |  |  |  |  |
| - Netherlands | 1.72 | 0.682 | 0.46, 6.77 | 0.425 |
| - Slovenia | 1.26 | 0.701 | 0.31, 5.02 | 0.740 |
| - Spain | 2.51 | 0.562 | 0.84, 7.74 | 0.102 |
| **Inclusion Season ‡** |  |  |  |  |
| - Spring | 0.94 | 0.577 | 0.30, 2.91 | 0.914 |
| - Summer | 0.65 | 0.621 | 0.19, 2.18 | 0.493 |
| - Winter | 1.31 | 0.668 | 0.35, 4.97 | 0.688 |
| **GINA Treatment step §** |  |  |  |  |
| - Step4 | 2.97 | 0.483 | 1.17, 7.88 | **0.024** |
| - Step5 | 17.0 | 1.14 | 2.56, 342 | **0.013** |
| **Current Smoking Exposure** | 0.79 | 0.448 | 0.33, 1.91 | 0.598 |

Footnote Table S5: Multi-variate logistic regression model showing the association between high bronchodilator response (BDR) according to the FEV₁ z-score definition and asthma control after adjusting for different confounders. Odds ratios (ORs) and 95% confidence intervals (CIs) for uncontrolled asthma are shown for each variable, with controlled asthma as the reference group. Variables include high bronchodilator response (BDR, defined as FEV₁ z-score increase >0.78 ), baseline FEV₁, country of inclusion, BMI z-score, season of inclusion, GINA treatment step, and current smoking exposure. Abbreviations: BDR = bronchodilator response; FEV₁= forced expiratory volume in one second; OR= odds ratio; CI= confidence interval; SE= standard error; BMI= body mass index; GINA= Global Initiative for Asthma. † Germany was selected randomly as a reference group for comparison of country of inclusion, ‡ Autumn was selected randomly as a reference group for comparison of Inclusion season, $ Step 3 was selected randomly as a reference group for comparison of GINA Treatment Step.


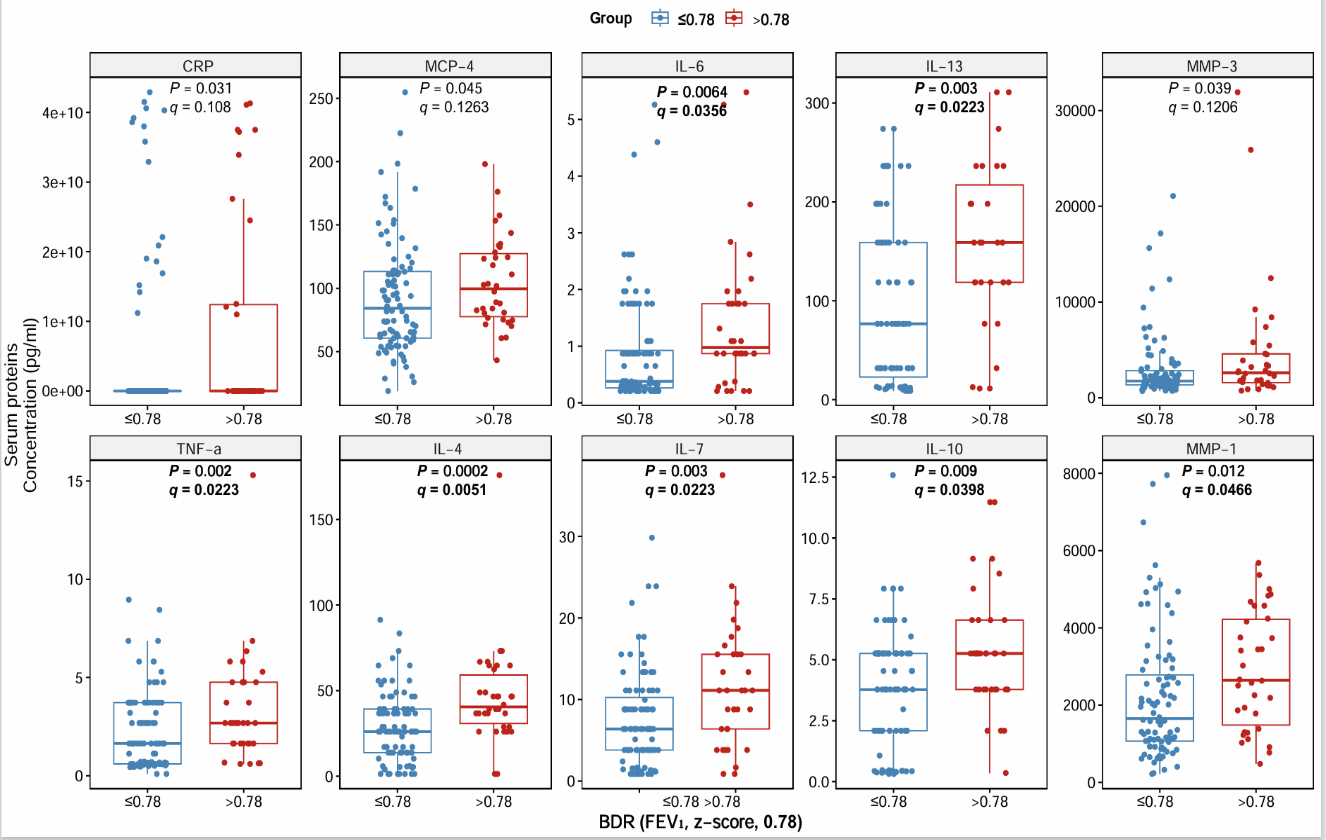


Figure S4: Boxplots of serum cytokine and protein expression in children with high BDR and low BDR, shown separately for BDR definition: FEV₁ z-score >0.78 (high BDR). Boxplots display the distribution of significantly different cytokines and proteins between groups. Blue boxes represent the low BDR group, while red boxes represent the high BDR group. Abbreviations: BDR = bronchodilator response; FEV₁ = forced expiratory volume in one second; CRP = C-reactive protein; IL = interleukin; MCP = monocyte chemoattractant protein; MMP = matrix metalloproteinase; TARC = thymus and activation regulated chemokine; TNF-α = tumor necrosis factor alpha.


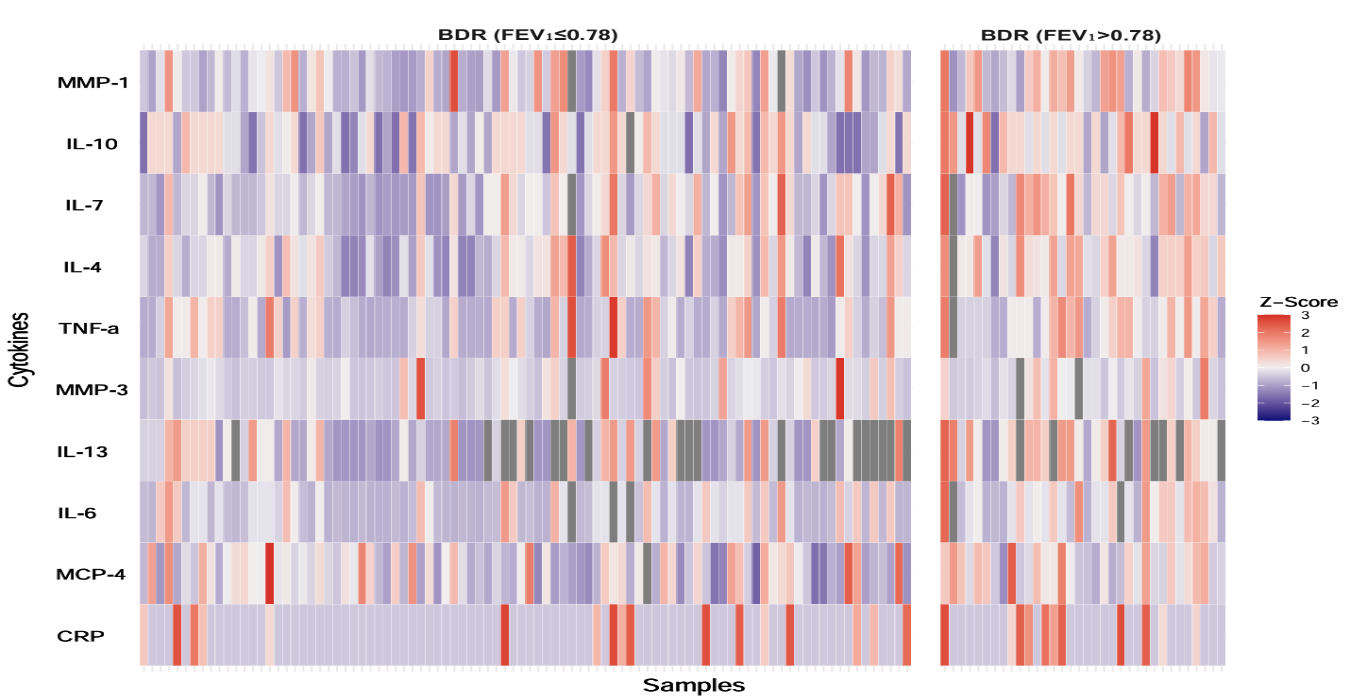


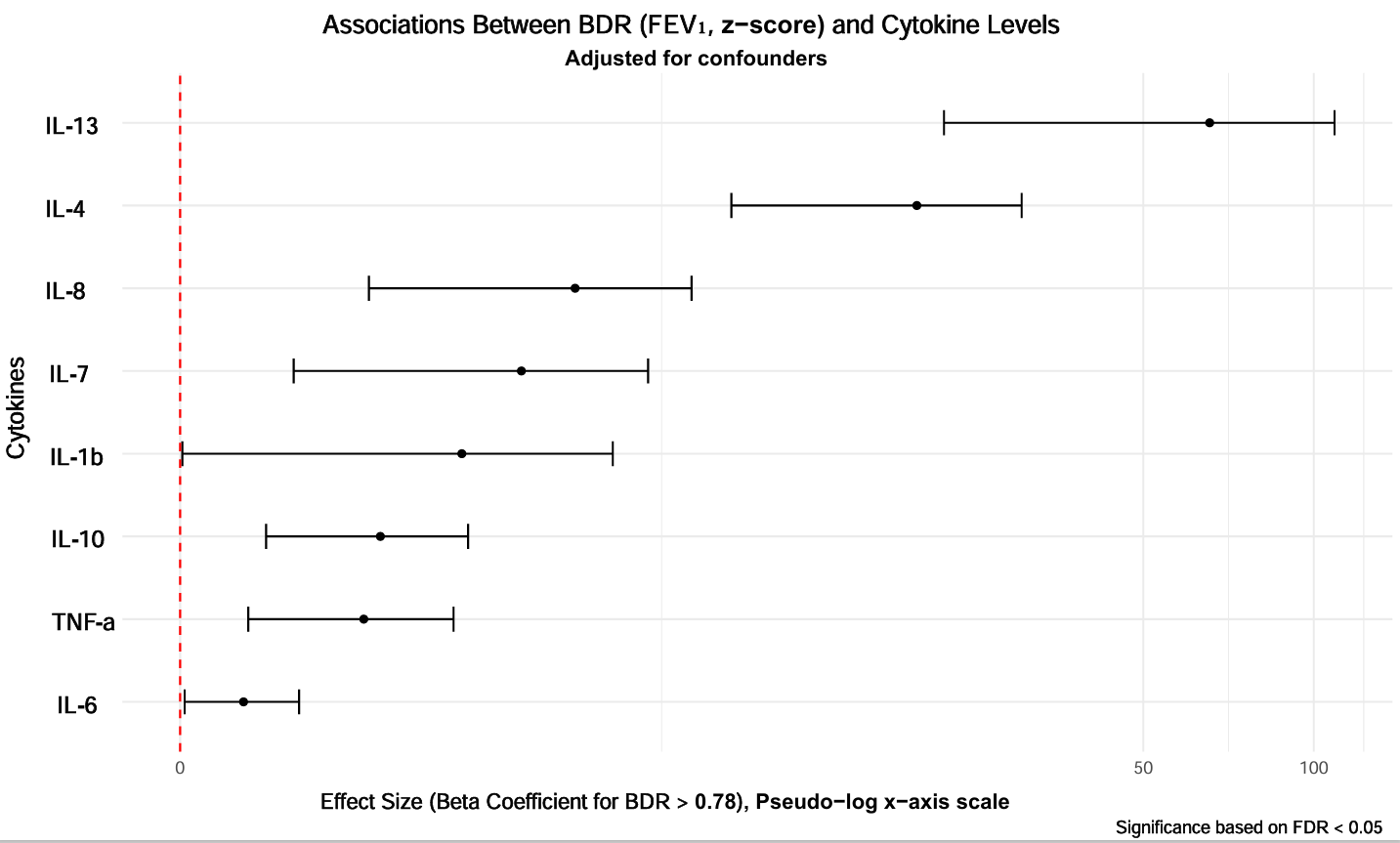
Figure S5: Heatmaps of serum cytokine and chemokines profiles in children with high and low BDR shown for FEV₁ z-score >0.78 (high BDR). Each row represents a cytokine or protein, and each column represents an individual participant. Color intensity reflects relative expression levels, illustrating the separation between high BDR and low BDR groups; CRP = C-reactive protein; IL = interleukin; MCP = monocyte chemoattractant protein; MMP = matrix metalloproteinase; TARC = thymus and activation regulated chemokine; TNF-α = tumor necrosis factor alpha.

Figure S6: Adjusted associations between high bronchodilator response (BDR) and serum proteins levels. Associations for high BDR defined as FEV₁ z-score >0.78. Beta coefficients (β) represent the direction and magnitude of association, with FDR-adjusted p-values indicating statistical significance. β coefficients are displayed as point estimates, with horizontal lines representing the corresponding 95% confidence intervals. All models were adjusted for age, sex, ethnicity, BMI z-score, GINA treatment step, baseline lung function, study site, season of inclusion, and current smoking exposure. *Abbreviations:* FEV₁ = forced expiratory volume in 1 second; BDR = bronchodilator response; IL = interleukin; TNF-α = tumor necrosis factor alpha; MMP = matrix metalloproteinase; FDR = false discovery rate. A pseudo-log X scale was used only for visualization pupose to allow plotting cytokines in different pg/ml ranges in one plot.


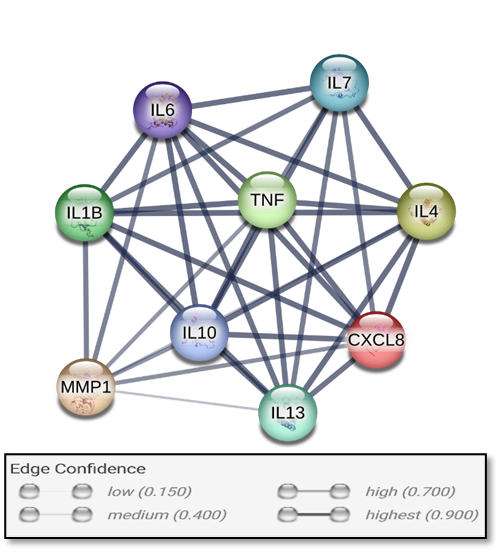


Figure S7: Protein-protein interaction network of high bronchodilator response (BDR) -associated cytokines and inflammatory markers using the STRING database.


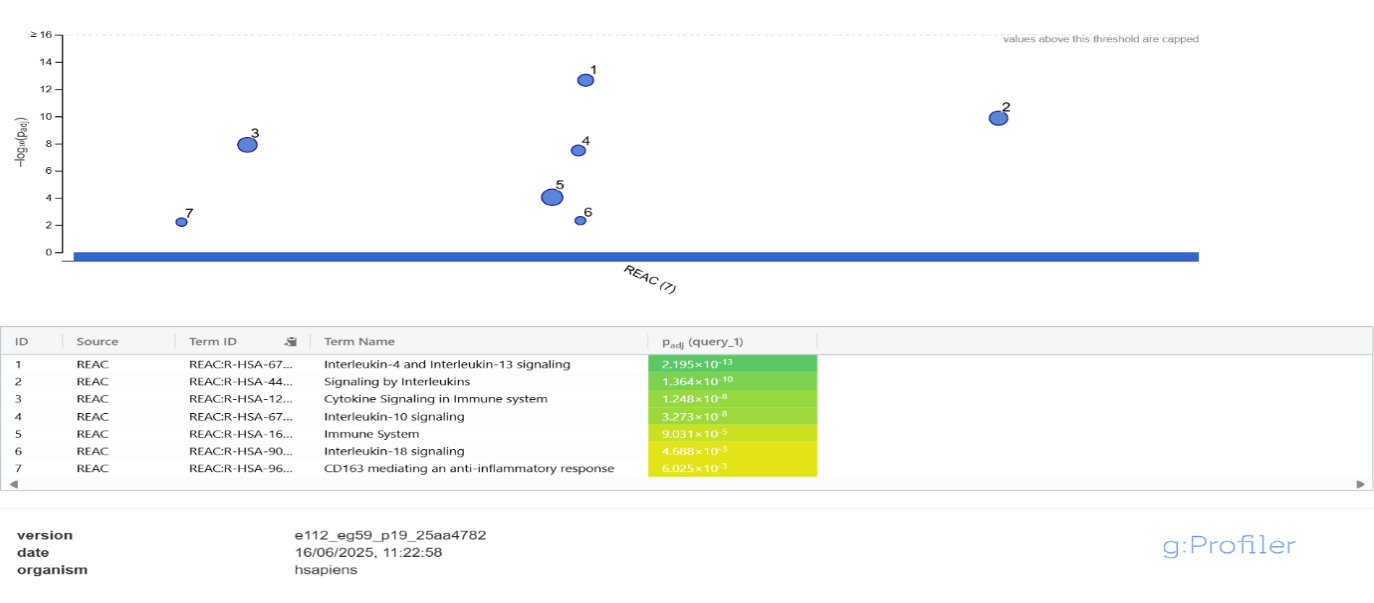

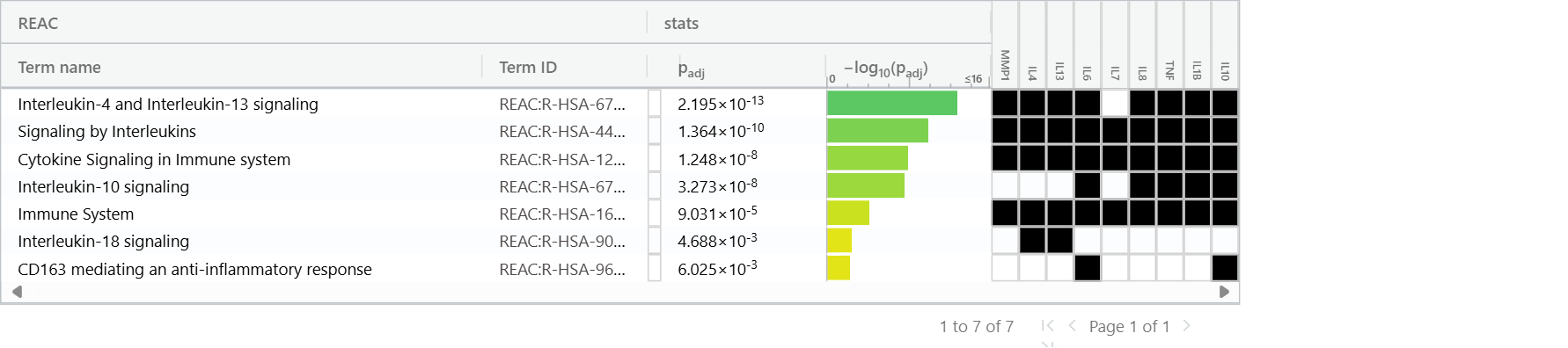


Figure S8: Pathway enrichment analysis of high BDR-associated cytokines and proteins using Reactome via g:Profiler. Bar plot displays the top Reactome (REAC) pathways significantly enriched among the high BDR-associated molecules (MMP-1, IL-4, IL-13, IL-6, IL-7, IL-8, IL-10, TNF, and IL-1β). The y-axis shows the –log₁₀(padj) values for each pathway, with higher values indicating greater statistical significance. Adjusted p-values (padj) were calculated to account for multiple testing using the default false discovery rate (FDR) correction method implemented in g:Profiler. Abbreviations: REAC = Reactome pathway database; padj = adjusted p-value; MMP – matrix metalloproteinase; IL – interleukin; TNF – tumor necrosis factor.

## 8. References

1. ATS/ERS recommendations for standardized procedures for the online and offline measurement of exhaled lower respiratory nitric oxide and nasal nitric oxide, 2005. Am J Respir Crit Care Med. 2005;171(8):912-30.

2. Alizadeh Bahmani AH, Vijverberg SJH, Hashimoto S, Wolff C, Almqvist C, Bloemsma LD, et al. Association of blood inflammatory phenotypes and asthma burden in children with moderate-to-severe asthma. ERJ Open Res. 2024;10(6).
