# Supplementary material for: Bronchodilator response is linked with uncontrolled moderate‐to‐severe childhood asthma and elevated IL‐4 and IL‐13
Source: Pediatr Allergy Immunol. 2026 Jun 8;37(6):e70392. doi: 10.1111/pai.70392 (PMC13244407; doi:10.1111/pai.70392)
Supplement: Supplementary file 1 — Table S1: Inflammatory markers measured by Luminex assay. Figure S1: Assessment of Bronchodilator Response (BDR) Using Spirometry. This figure illustrates the spirometry procedure, where a patient performs inhalation and exhalation to measure lung function. The upper section represents the pre‐bronchodilator state, showing airway constriction and reduced airflow. The lower section represents the post‐bronchodilator state, where bronchodilator administration leads to airway relaxation and improved airflow. The graphs highlight Forced Expiratory Volume in 1 s (FEV1), a key parameter that measures the air breathed out forcefully within the first second after a deep breath. A significant improvement in FEV1 post‐bronchodilator is often used to confirm conditions like asthma. Figure S2: Directed Acyclic Graph (DAG) illustrating the potential confounders considered in the analysis of the relationship between bronchodilator response (BDR), asthma control, and serum cytokines/chemokines. The DAG identifies key variables, including age, sex, ethnicity, BMI z‐score, baseline lung function, country, season of inclusion, GINA step, and current smoking exposure, guiding appropriate adjustment in statistical models to minimize confounding bias. Figure S3: Overlap of high BDR classifications according to the two BDR definitions (>10% predicted and z‐score >0.78). All children identified by the >10% definition were also included in the z‐score group, with 8 additional children classified only by the z‐score. Table S2: (A) Demographic and clinical characteristics of children with high and low BDR according to the FEV1 z‐score definition. (B) Lung function, white blood cell counts, and atopic sensitization in children with high and low BDR according to the FEV1 z‐score definition. (C) Asthma medication use and treatment steps in children with high and low BDR according to the FEV1 z‐score definition. Table S3: Multi‐variate logistic regression model showing the association between BD [file PAI-37-e70392-s001.zip › SysPharmPediA consortium list_15-09-2022.docx]

**SysPharmPediA consortium list**

The SysPharmPediA consortium wishes to acknowledge the help and expertise of the following individuals and groups without whom, the study would not have been possible.

**SysPharmPediA members:**

| **Name:** | **Country:** | **Affiliation:** |
| --- | --- | --- |
| Olaia Sardón-Prado MD, PhD | Spain | Division of Pediatric Respiratory Medicine, Hospital Universitario Donostia, San Sebastián, Spain  Department of Pediatrics, University of the Basque Country (UPV/EHU), San Sebastián, Spain. |
| Paula Corcuera-Elósegui MD, phD | Spain | Division of Pediatric Respiratory Medicine, Hospital Universitario Donostia, San Sebastián, Spain. |
| Javier Korta-Murua MD, PhD | Spain | Division of Pediatric Respiratory Medicine, Hospital Universitario Donostia, San Sebastián, Spain  Department of Pediatrics, University of the Basque Country (UPV/EHU), San Sebastián, Spain. |
| Mahmoud I. Abdel-Aziz PhD | The Netherlands | Department of Respiratory Medicine, Amsterdam UMC, University of Amsterdam, Amsterdam, The Netherlands.  Department of Clinical Pharmacy, Faculty of Pharmacy, Assiut University, Assiut, Egypt. |
| Paul Brinkman PhD | The Netherlands | Department of Respiratory Medicine, Amsterdam UMC, University of Amsterdam, Amsterdam, The Netherlands. |
| Susanne J. H. Vijverberg PhD | The Netherlands | Department of Respiratory Medicine, Amsterdam UMC, University of Amsterdam, Amsterdam, The Netherlands. |
| Anne H. Neerincx PhD | The Netherlands | Department of Respiratory Medicine, Amsterdam UMC, University of Amsterdam, Amsterdam, The Netherlands. |
| Simone Hashimoto MD PhD | The Netherlands | Department of Respiratory Medicine, Amsterdam UMC, University of Amsterdam, Amsterdam, The Netherlands.  Department of Pediatric Respiratory Medicine, Emma Children's Hospital, Amsterdam UMC, Amsterdam, The Netherlands. |
| Aletta D. Kraneveld PhD | The Netherlands | Division of Pharmacology, Utrecht Institute for Pharmaceutical Sciences, Faculty of Science, Utrecht University, Utrecht, the Netherlands.  Institute for Risk Assessment Sciences, Faculty of Veterinary Medicine, Utrecht University, Utrecht, the Netherlands. |
| Anke H. Maitland-van der Zee PharmD, PhD | The Netherlands | Department of Respiratory Medicine, Amsterdam UMC, University of Amsterdam, Amsterdam, The Netherlands.  Department of Pediatric Respiratory Medicine, Emma Children's Hospital, Amsterdam UMC, Amsterdam, The Netherlands. |
| Maria Pino-Yanes, PhD | Spain - Tenerife | Genomics and Health Group, Department of Biochemistry, Microbiology, Cell Biology and Genetics, Universidad de La Laguna, La Laguna, Santa Cruz de Tenerife, Spain.  CIBER de Enfermedades Respiratorias, Instituto de Salud Carlos III, Madrid, Spain.  Instituto de Tecnologías Biomédicas (ITB), Universidad de La Laguna, Santa Cruz de Tenerife, Spain. |
| Natalia Hernandez-Pacheco, MSc | Spain - Tenerife | Research Unit, Hospital Universitario N.S. de Candelaria, Universidad de La Laguna, Santa Cruz de Tenerife, Spain  Genomics and Health Group, Department of Biochemistry, Microbiology, Cell Biology and Genetics, Universidad de La Laguna, La Laguna, Santa Cruz de Tenerife, Spain. |
| Esther Herrera-Luis, MSc | Spain - Tenerife | Genomics and Health Group, Department of Biochemistry, Microbiology, Cell Biology and Genetics, Universidad de La Laguna, La Laguna, Santa Cruz de Tenerife, Spain. |
| Javier Perez-Garcia, BS | Spain - Tenerife | Genomics and Health Group, Department of Biochemistry, Microbiology, Cell Biology and Genetics, Universidad de La Laguna, La Laguna, Santa Cruz de Tenerife, Spain. |
| Fabian Lorenzo-Diaz, PhD | Spain - Tenerife | Genomics and Health Group, Department of Biochemistry, Microbiology, Cell Biology and Genetics, Universidad de La Laguna, La Laguna, Santa Cruz de Tenerife, Spain. |
| Uroš Potočnik, PhD | Slovenia | Center for Human Molecular Genetics and Pharmacogenomics, Faculty of Medicine, University of Maribor, Maribor, Slovenia.  Laboratory for Biochemistry, Molecular biology and Genomics, Faculty of Chemistry and Chemical Engineering, University of Maribor, Maribor, Slovenia.  Department for Science and Research,University Medical Centre Maribor, Maribor, Slovenia |
| Mario Gorenjak MBI, PhD | Slovenia | Center for Human Molecular Genetics and Pharmacogenomics, Faculty of Medicine, University of Maribor, Maribor, Slovenia. |
| Vojko Berce MD, PhD | Slovenia | Center for Human Molecular Genetics and Pharmacogenomics, Faculty of Medicine, University of Maribor, Maribor, Slovenia.  Clinic of Pediatrics, University Medical Centre Maribor, Maribor, Slovenia. |
| Maya Petek, PhD | Slovenia | Center for Human Molecular Genetics and Pharmacogenomics, Faculty of Medicine, University of Maribor, Maribor, Slovenia. |
| Michael Kabesch MD, PhD | Germany | Department of Pediatric Pneumology and Allergy, University Children’s Hospital Regensburg (KUNO) at the Hospital St. Hedwig of the Order of St. John, University of Regensburg, Regensburg, Germany.  Science and Development Campus Regensburg (WECARE), University Children’s Hospital Regensburg (KUNO) at the Hospital St. Hedwig of the Order of St. John, University of Regensburg, Regensburg, Germany. |
| Susanne Harner | Germany | Department of Pediatric Pneumology and Allergy, University Children’s Hospital Regensburg (KUNO) at the Hospital St. Hedwig of the Order of St. John, University of Regensburg, Regensburg, Germany. |
| Christine Wolff | Germany | Science and Development Campus Regensburg (WECARE), University Children’s Hospital Regensburg (KUNO) at the Hospital St. Hedwig of the Order of St. John, University of Regensburg, Regensburg, Germany |
| Antoaneta Toncheva | Germany | Department of Pediatric Pneumology and Allergy, University Children’s Hospital Regensburg (KUNO) at the Hospital St. Hedwig of the Order of St. John, University of Regensburg, Regensburg, Germany. |
| Susanne Brandstetter | Germany | Science and Development Campus Regensburg (WECARE), University Children’s Hospital Regensburg (KUNO) at the Hospital St. Hedwig of the Order of St. John, University of Regensburg, Regensburg, Germany. |
| Elisa Valletta | Germany | Science and Development Campus Regensburg (WECARE), University Children’s Hospital Regensburg (KUNO) at the Hospital St. Hedwig of the Order of St. John, University of Regensburg, Regensburg, Germany. |
| Heike Buntrock-Döpke | Germany | Science and Development Campus Regensburg (WECARE), University Children’s Hospital Regensburg (KUNO) at the Hospital St. Hedwig of the Order of St. John, University of Regensburg, Regensburg, Germany. |
| Joris C. Verster PhD | The Netherlands | Division of Pharmacology, Utrecht Institute for Pharmaceutical Sciences, Faculty of Science, Utrecht University, Utrecht, the Netherlands.  Institute for Risk Assessment Sciences, Faculty of Veterinary Medicine, Utrecht University, Utrecht, the Netherlands. |
| Nikki Kerssemakers | The Netherlands | Division of Pharmacology, Utrecht Institute for Pharmaceutical Sciences, Faculty of Science, Utrecht University, Utrecht, the Netherlands. |
| Catarina Almqvist MD, PhD | Sweden | Dept of Medical Epidemiology and Biostatistics, Karolinska Institutet, Stockholm, Sweden.  Pediatric Allergy and Pulmonology Unit at Astrid Lindgren Children’s Hospital, Karolinska University Hospital. |
| Anna Hedman PhD | Sweden | Dept of Medical Epidemiology and Biostatistics, Karolinska Institutet, Stockholm, Sweden. |
| Mwenya Mubanga PhD | Sweden | Dept of Medical Epidemiology and Biostatistics, Karolinska Institutet, Stockholm, Sweden. |
| Tong Gong PhD | Sweden | Dept of Medical Epidemiology and Biostatistics, Karolinska Institutet, Stockholm, Sweden. |
| Anne Örtqvist | Sweden | Dept of Medical Epidemiology and Biostatistics, Karolinska Institutet, Stockholm, Sweden. |

**Others that contributed to SysPharmPediA:**

| **Name:** | **Country:** | **Affiliation:** |
| --- | --- | --- |
| Amaia Lorea Alvarez RN | Spain | Division of Pediatric Respiratory Medicine, Hospital Universitario Donostia, San Sebastián, Spain. |
| Arantzazu Zugasti Pérez RN | Spain | Division of Pediatric Respiratory Medicine, Hospital Universitario Donostia, San Sebastián, Spain. |
| Miren Lorea Otero Aramburu, NA | Spain | Division of Pediatric Respiratory Medicine, Hospital Universitario Donostia, San Sebastián, Spain. |
| María Isabel Gomez Osua RN^3^ | Spain | Basque Biobank (Basque Biobank), Biodonostia Health Research Institute Association Node [www.biobancovasco.org](http://www.biobancovasco.org) |
| María Isabel Gomez Osua RN^3^ | Spain | Basque Biobank (Basque Biobank), Biodonostia Health Research Institute Association Node [www.biobancovasco.org](http://www.biobancovasco.org) |
| Yennece W.F. Dagelet | The Netherlands | Department of Respiratory Medicine, Amsterdam UMC, University of Amsterdam, Amsterdam, The Netherlands. |
| Suzanne Terheggen-Lagro MD, PhD | The Netherlands | Department of Pediatric Respiratory Medicine, Emma Children's Hospital, Amsterdam UMC, Amsterdam, The Netherlands. |
| Niels W.P. Rutjes MD | The Netherlands | Department of Pediatric Respiratory Medicine, Emma Children's Hospital, Amsterdam UMC, Amsterdam, The Netherlands. |
| E. G. Haarman | The Netherlands | Department of Pediatric Respiratory Medicine, Emma Children's Hospital, Amsterdam UMC, Amsterdam, The Netherlands. |
| T. Dekker | The Netherlands | Department of Experimental Immunology, Academic Medical Center (AMC), University of Amsterdam, Amsterdam, The Netherlands. |
| B.S Dierdorp | The Netherlands | Department of Experimental Immunology, Academic Medical Center (AMC), University of Amsterdam, Amsterdam, The Netherlands. |
| R. Khurshid | The Netherlands | Department of Experimental Immunology, Academic Medical Center (AMC), University of Amsterdam, Amsterdam, The Netherlands. |
| B. Kapitein, MD, PhD | The Netherlands | Paediatric Intensive Care, Emma Children's Hospital, Amsterdam UMC, Amsterdam, The Netherlands. |
| G. Biesbroek | The Netherlands | Department of Pediatric Respiratory Medicine, Emma Children's Hospital, Amsterdam UMC, Amsterdam, The Netherlands. |
| J. Israels | The Netherlands | Department of Pediatric Respiratory Medicine, Emma Children's Hospital, Amsterdam UMC, Amsterdam, The Netherlands. |
| C. Brackel | The Netherlands | Department of Pediatric Respiratory Medicine, Emma Children's Hospital, Amsterdam UMC, Amsterdam, The Netherlands. |
| M. Verwaal – Maasstad | The Netherlands | Department of Pediatrics, Maasstad Hospital, Rotterdam, The Netherlands. |
| D. Seljogi | The Netherlands | Department of Pediatric Respiratory Medicine, Emma Children's Hospital, Amsterdam UMC, Amsterdam, The Netherlands. |
| P.F.M. Mau Asam | The Netherlands | Department of Respiratory Medicine, Amsterdam UMC, University of Amsterdam, Amsterdam, The Netherlands. |
| S. J.A. Lone-Latif | The Netherlands | Department of Respiratory Medicine, Amsterdam UMC, University of Amsterdam, Amsterdam, The Netherlands. |
| Davey Stigters | The Netherlands | Utrecht University, Master Pharmacy, Utrecht, The Netherlands. |
| Emine Sariguney | The Netherlands | Department of Respiratory Medicine, Amsterdam UMC, University of Amsterdam, Amsterdam, The Netherlands. |
| André Franke | Germany | Kiel |
| Juha Kere | Sweden/Finland | Stockholm/Helsinki |
| Anne Örtqvist | Sweden | Dept of Medical Epidemiology and Biostatistics, Karolinska Institutet, Stockholm, Sweden. |
| Vilhelmina Ullemar | Sweden | Dept of Medical Epidemiology and Biostatistics, Karolinska Institutet, Stockholm, Sweden. |
| Karin Dellenvall | Sweden | Dept of Medical Epidemiology and Biostatistics, Karolinska Institutet, Stockholm, Sweden. |
| Amir Hossein Alizadeh Bahmani PharmD | The Netherlands | Amsterdam UMC location University of Amsterdam, Department of Pulmonary Medicine, Meibergdreef 9, Amsterdam, the Netherlands |
| Jelle M. Blankestijn MSc | The Netherlands | Amsterdam UMC location University of Amsterdam, Department of Pulmonary Medicine, Meibergdreef 9, Amsterdam, the Netherlands |
| Shahriyar Shahbazi Khamas PharmD | The Netherlands | Amsterdam UMC location University of Amsterdam, Department of Pulmonary Medicine, Meibergdreef 9, Amsterdam, the Netherlands |

**Funding:**

The SysPharmPediA consortium is supported by ZonMW [project number: 9003035001], the Ministry of Education, Science, and Sport of the Republic of Slovenia [contract number C330-16-500106]; the German Ministry of Education and Research (BMBF) [project number FKZ 031L0088]; Instituto de Salud Carlos III (ISCIII) through Strategic Action for Health Research (AES) and European Community (EC) within the Active and Assisted Living (AAL) Program framework [award numbers AC15/00015 and AC15/00058] under the frame of the ERACoSysMed JTC-1 Call. M.P.-Y. was funded by the Ramón y Cajal Program (RYC-2015-17205) by the Spanish Ministry of Science and Innovation (MICINN), the State Research Agency, and the European Re-gional Development Fund from the European Union (MICINN/AEI/FEDER, UE, grant SAF2017-83417R). J.P.-G. was supported by a Ph.D. fellowship (FPU19/02175) granted by MICINN. U.P. and M.G. were funded by Slovenian Research Agency (research core funding No. P3-0067). M.I.A.-A. was funded by the Egyptian Government Ph.D. Scholarships. The STOPPA study was funded by the Swedish Research Council project grant 2018-02640 and the Swedish Asthma and Allergy Research Foundation.

**Input Spain:**

Human samples and related data used in this study were provided by the Basque Biobank/Biodonostia Node [www.biobancovasco.org](http://www.biobancovasco.org) and were processed following standard operating procedures with appropriate approval of the relevant Ethics Committee and Scientific Advisory Board.
