# Supplementary figures and images for: Bronchodilator response is linked with uncontrolled moderate‐to‐severe childhood asthma and elevated IL‐4 and IL‐13
Source: Pediatr Allergy Immunol. 2026 Jun 8;37(6):e70392. doi: 10.1111/pai.70392 (PMC13244407; doi:10.1111/pai.70392)

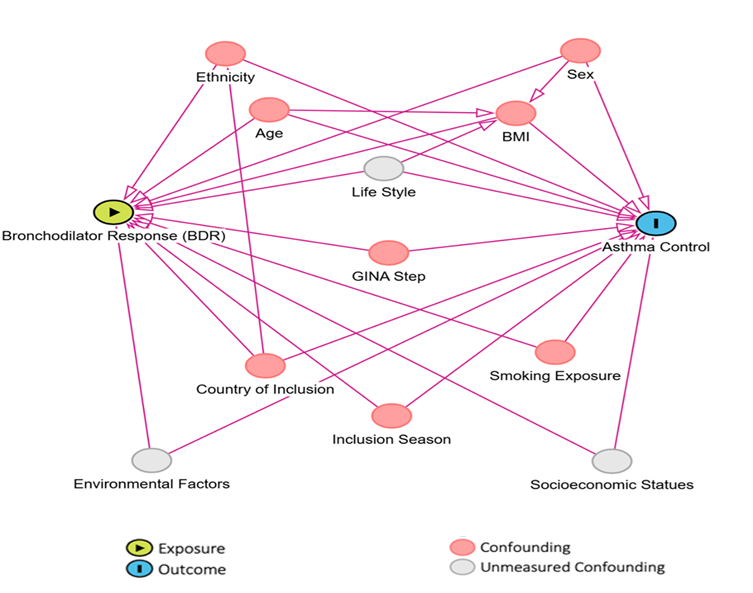

Supplement: Supplementary file 1 — Table S1: Inflammatory markers measured by Luminex assay. Figure S1: Assessment of Bronchodilator Response (BDR) Using Spirometry. This figure illustrates the spirometry procedure, where a patient performs inhalation and exhalation to measure lung function. The upper section represents the pre‐bronchodilator state, showing airway constriction and reduced airflow. The lower section represents the post‐bronchodilator state, where bronchodilator administration leads to airway relaxation and improved airflow. The graphs highlight Forced Expiratory Volume in 1 s (FEV1), a key parameter that measures the air breathed out forcefully within the first second after a deep breath. A significant improvement in FEV1 post‐bronchodilator is often used to confirm conditions like asthma. Figure S2: Directed Acyclic Graph (DAG) illustrating the potential confounders considered in the analysis of the relationship between bronchodilator response (BDR), asthma control, and serum cytokines/chemokines. The DAG identifies key variables, including age, sex, ethnicity, BMI z‐score, baseline lung function, country, season of inclusion, GINA step, and current smoking exposure, guiding appropriate adjustment in statistical models to minimize confounding bias. Figure S3: Overlap of high BDR classifications according to the two BDR definitions (>10% predicted and z‐score >0.78). All children identified by the >10% definition were also included in the z‐score group, with 8 additional children classified only by the z‐score. Table S2: (A) Demographic and clinical characteristics of children with high and low BDR according to the FEV1 z‐score definition. (B) Lung function, white blood cell counts, and atopic sensitization in children with high and low BDR according to the FEV1 z‐score definition. (C) Asthma medication use and treatment steps in children with high and low BDR according to the FEV1 z‐score definition. Table S3: Multi‐variate logistic regression model showing the association between BD [file PAI-37-e70392-s001.zip › Figure S2.png]

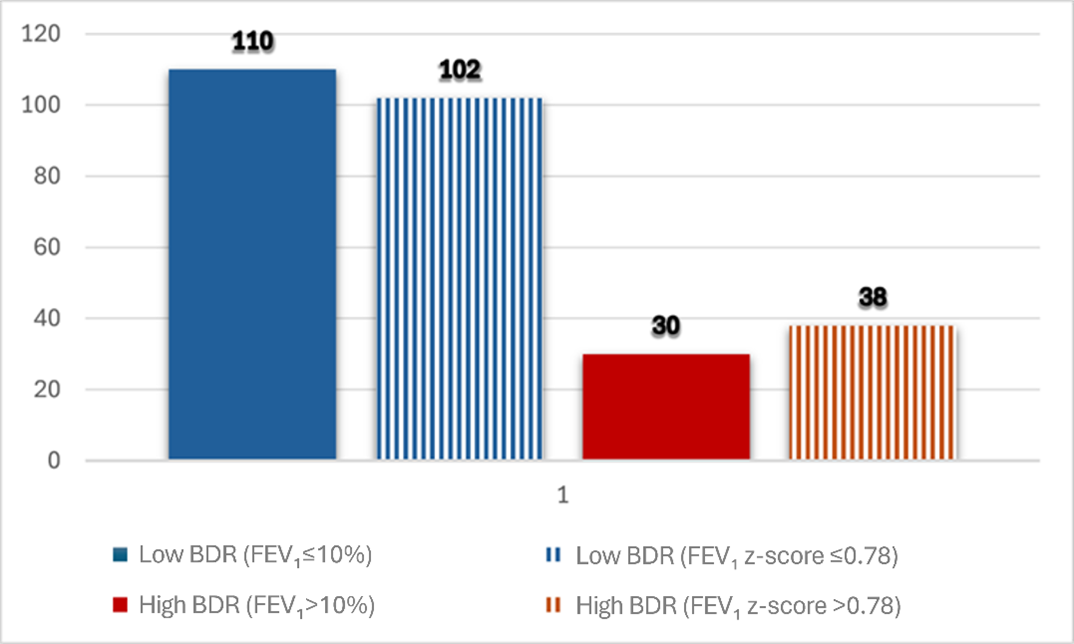

Supplement: Supplementary file 1 — Table S1: Inflammatory markers measured by Luminex assay. Figure S1: Assessment of Bronchodilator Response (BDR) Using Spirometry. This figure illustrates the spirometry procedure, where a patient performs inhalation and exhalation to measure lung function. The upper section represents the pre‐bronchodilator state, showing airway constriction and reduced airflow. The lower section represents the post‐bronchodilator state, where bronchodilator administration leads to airway relaxation and improved airflow. The graphs highlight Forced Expiratory Volume in 1 s (FEV1), a key parameter that measures the air breathed out forcefully within the first second after a deep breath. A significant improvement in FEV1 post‐bronchodilator is often used to confirm conditions like asthma. Figure S2: Directed Acyclic Graph (DAG) illustrating the potential confounders considered in the analysis of the relationship between bronchodilator response (BDR), asthma control, and serum cytokines/chemokines. The DAG identifies key variables, including age, sex, ethnicity, BMI z‐score, baseline lung function, country, season of inclusion, GINA step, and current smoking exposure, guiding appropriate adjustment in statistical models to minimize confounding bias. Figure S3: Overlap of high BDR classifications according to the two BDR definitions (>10% predicted and z‐score >0.78). All children identified by the >10% definition were also included in the z‐score group, with 8 additional children classified only by the z‐score. Table S2: (A) Demographic and clinical characteristics of children with high and low BDR according to the FEV1 z‐score definition. (B) Lung function, white blood cell counts, and atopic sensitization in children with high and low BDR according to the FEV1 z‐score definition. (C) Asthma medication use and treatment steps in children with high and low BDR according to the FEV1 z‐score definition. Table S3: Multi‐variate logistic regression model showing the association between BD [file PAI-37-e70392-s001.zip › Figure S3.png]

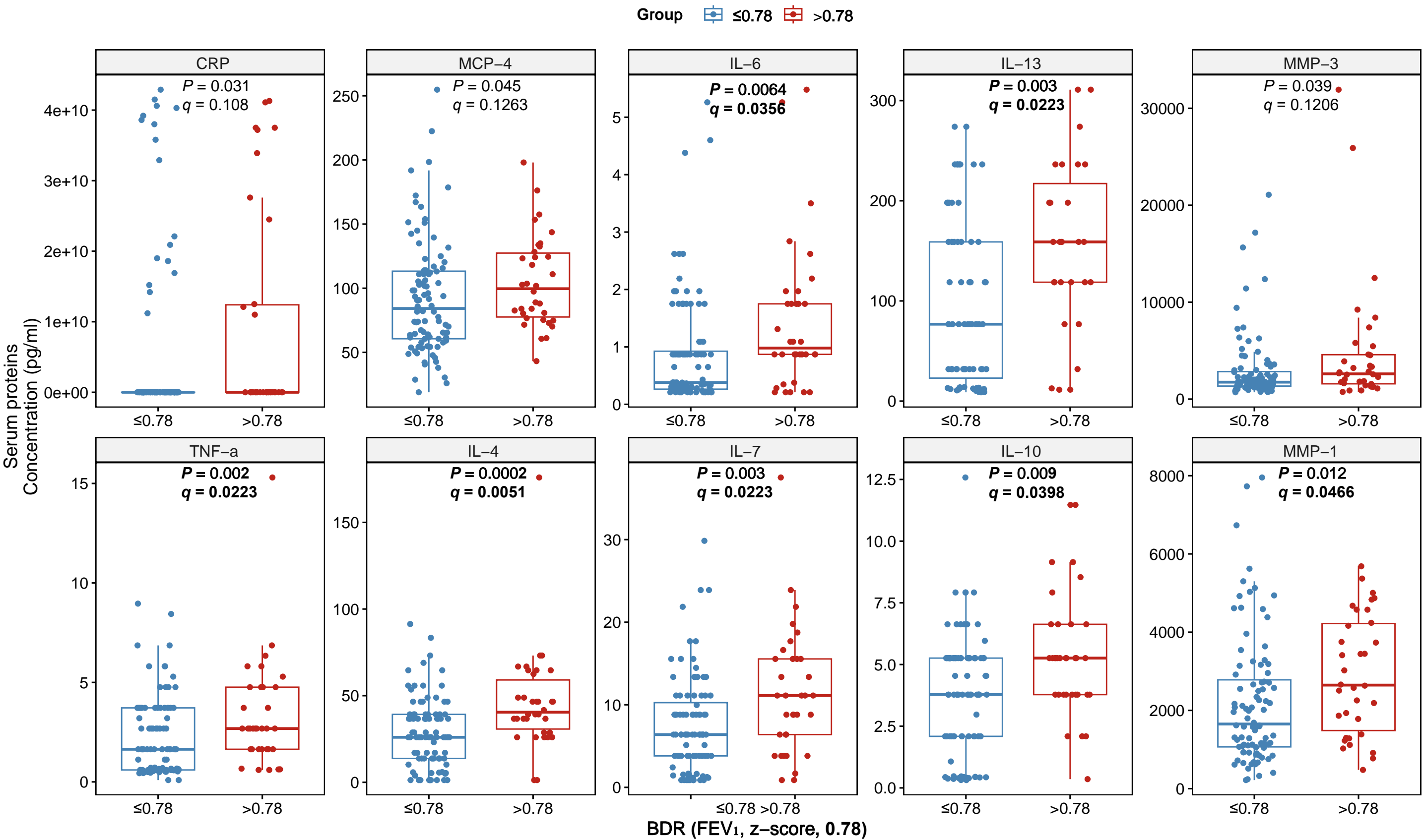

Supplement: Supplementary file 1 — Table S1: Inflammatory markers measured by Luminex assay. Figure S1: Assessment of Bronchodilator Response (BDR) Using Spirometry. This figure illustrates the spirometry procedure, where a patient performs inhalation and exhalation to measure lung function. The upper section represents the pre‐bronchodilator state, showing airway constriction and reduced airflow. The lower section represents the post‐bronchodilator state, where bronchodilator administration leads to airway relaxation and improved airflow. The graphs highlight Forced Expiratory Volume in 1 s (FEV1), a key parameter that measures the air breathed out forcefully within the first second after a deep breath. A significant improvement in FEV1 post‐bronchodilator is often used to confirm conditions like asthma. Figure S2: Directed Acyclic Graph (DAG) illustrating the potential confounders considered in the analysis of the relationship between bronchodilator response (BDR), asthma control, and serum cytokines/chemokines. The DAG identifies key variables, including age, sex, ethnicity, BMI z‐score, baseline lung function, country, season of inclusion, GINA step, and current smoking exposure, guiding appropriate adjustment in statistical models to minimize confounding bias. Figure S3: Overlap of high BDR classifications according to the two BDR definitions (>10% predicted and z‐score >0.78). All children identified by the >10% definition were also included in the z‐score group, with 8 additional children classified only by the z‐score. Table S2: (A) Demographic and clinical characteristics of children with high and low BDR according to the FEV1 z‐score definition. (B) Lung function, white blood cell counts, and atopic sensitization in children with high and low BDR according to the FEV1 z‐score definition. (C) Asthma medication use and treatment steps in children with high and low BDR according to the FEV1 z‐score definition. Table S3: Multi‐variate logistic regression model showing the association between BD [file PAI-37-e70392-s001.zip › Figure S4.pdf]

Cytokines

BDR (FEV<sub>1</sub>≤0.78)

BDR (FEV<sub>1</sub>>0.78)

MMP-1

IL-10

IL-7

IL-4

TNF-α

MMP-3

IL-13

IL-6

MCP-4

CRP

Samples

Z-Score

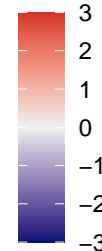

Supplement: Supplementary file 1 — Table S1: Inflammatory markers measured by Luminex assay. Figure S1: Assessment of Bronchodilator Response (BDR) Using Spirometry. This figure illustrates the spirometry procedure, where a patient performs inhalation and exhalation to measure lung function. The upper section represents the pre‐bronchodilator state, showing airway constriction and reduced airflow. The lower section represents the post‐bronchodilator state, where bronchodilator administration leads to airway relaxation and improved airflow. The graphs highlight Forced Expiratory Volume in 1 s (FEV1), a key parameter that measures the air breathed out forcefully within the first second after a deep breath. A significant improvement in FEV1 post‐bronchodilator is often used to confirm conditions like asthma. Figure S2: Directed Acyclic Graph (DAG) illustrating the potential confounders considered in the analysis of the relationship between bronchodilator response (BDR), asthma control, and serum cytokines/chemokines. The DAG identifies key variables, including age, sex, ethnicity, BMI z‐score, baseline lung function, country, season of inclusion, GINA step, and current smoking exposure, guiding appropriate adjustment in statistical models to minimize confounding bias. Figure S3: Overlap of high BDR classifications according to the two BDR definitions (>10% predicted and z‐score >0.78). All children identified by the >10% definition were also included in the z‐score group, with 8 additional children classified only by the z‐score. Table S2: (A) Demographic and clinical characteristics of children with high and low BDR according to the FEV1 z‐score definition. (B) Lung function, white blood cell counts, and atopic sensitization in children with high and low BDR according to the FEV1 z‐score definition. (C) Asthma medication use and treatment steps in children with high and low BDR according to the FEV1 z‐score definition. Table S3: Multi‐variate logistic regression model showing the association between BD [file PAI-37-e70392-s001.zip › Figure S5.pdf]

# Associations Between BDR (FEV<sub>1</sub>, z-score) and Cytokine Levels

Adjusted for confounders

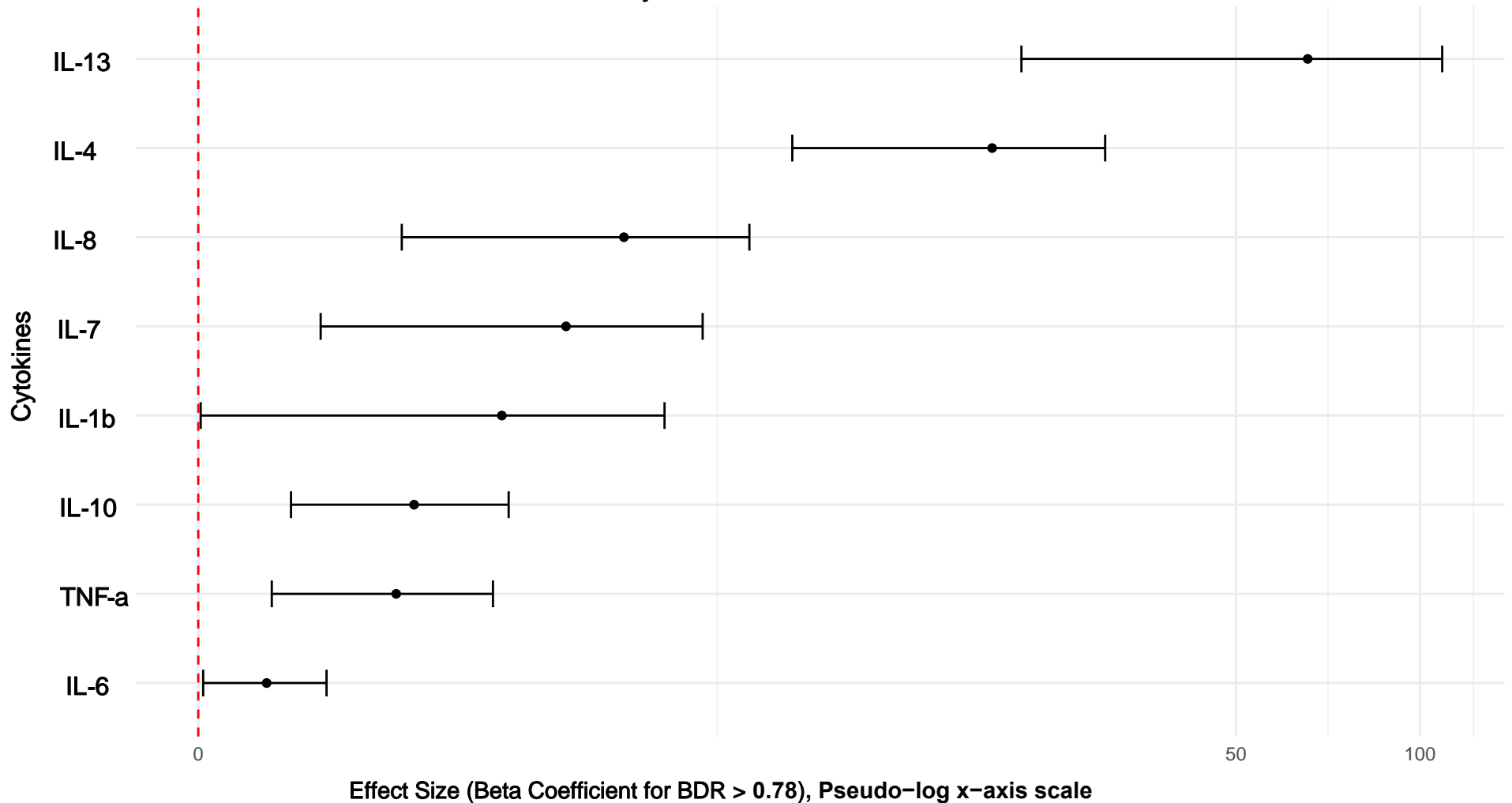

Supplement: Supplementary file 1 — Table S1: Inflammatory markers measured by Luminex assay. Figure S1: Assessment of Bronchodilator Response (BDR) Using Spirometry. This figure illustrates the spirometry procedure, where a patient performs inhalation and exhalation to measure lung function. The upper section represents the pre‐bronchodilator state, showing airway constriction and reduced airflow. The lower section represents the post‐bronchodilator state, where bronchodilator administration leads to airway relaxation and improved airflow. The graphs highlight Forced Expiratory Volume in 1 s (FEV1), a key parameter that measures the air breathed out forcefully within the first second after a deep breath. A significant improvement in FEV1 post‐bronchodilator is often used to confirm conditions like asthma. Figure S2: Directed Acyclic Graph (DAG) illustrating the potential confounders considered in the analysis of the relationship between bronchodilator response (BDR), asthma control, and serum cytokines/chemokines. The DAG identifies key variables, including age, sex, ethnicity, BMI z‐score, baseline lung function, country, season of inclusion, GINA step, and current smoking exposure, guiding appropriate adjustment in statistical models to minimize confounding bias. Figure S3: Overlap of high BDR classifications according to the two BDR definitions (>10% predicted and z‐score >0.78). All children identified by the >10% definition were also included in the z‐score group, with 8 additional children classified only by the z‐score. Table S2: (A) Demographic and clinical characteristics of children with high and low BDR according to the FEV1 z‐score definition. (B) Lung function, white blood cell counts, and atopic sensitization in children with high and low BDR according to the FEV1 z‐score definition. (C) Asthma medication use and treatment steps in children with high and low BDR according to the FEV1 z‐score definition. Table S3: Multi‐variate logistic regression model showing the association between BD [file PAI-37-e70392-s001.zip › Figure S6.pdf]

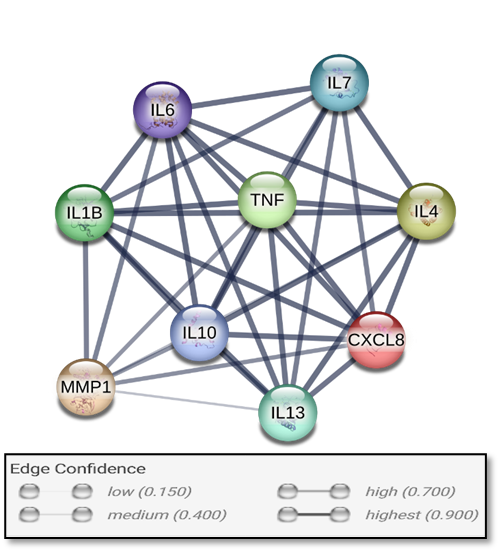

Supplement: Supplementary file 1 — Table S1: Inflammatory markers measured by Luminex assay. Figure S1: Assessment of Bronchodilator Response (BDR) Using Spirometry. This figure illustrates the spirometry procedure, where a patient performs inhalation and exhalation to measure lung function. The upper section represents the pre‐bronchodilator state, showing airway constriction and reduced airflow. The lower section represents the post‐bronchodilator state, where bronchodilator administration leads to airway relaxation and improved airflow. The graphs highlight Forced Expiratory Volume in 1 s (FEV1), a key parameter that measures the air breathed out forcefully within the first second after a deep breath. A significant improvement in FEV1 post‐bronchodilator is often used to confirm conditions like asthma. Figure S2: Directed Acyclic Graph (DAG) illustrating the potential confounders considered in the analysis of the relationship between bronchodilator response (BDR), asthma control, and serum cytokines/chemokines. The DAG identifies key variables, including age, sex, ethnicity, BMI z‐score, baseline lung function, country, season of inclusion, GINA step, and current smoking exposure, guiding appropriate adjustment in statistical models to minimize confounding bias. Figure S3: Overlap of high BDR classifications according to the two BDR definitions (>10% predicted and z‐score >0.78). All children identified by the >10% definition were also included in the z‐score group, with 8 additional children classified only by the z‐score. Table S2: (A) Demographic and clinical characteristics of children with high and low BDR according to the FEV1 z‐score definition. (B) Lung function, white blood cell counts, and atopic sensitization in children with high and low BDR according to the FEV1 z‐score definition. (C) Asthma medication use and treatment steps in children with high and low BDR according to the FEV1 z‐score definition. Table S3: Multi‐variate logistic regression model showing the association between BD [file PAI-37-e70392-s001.zip › Figure S7.png]

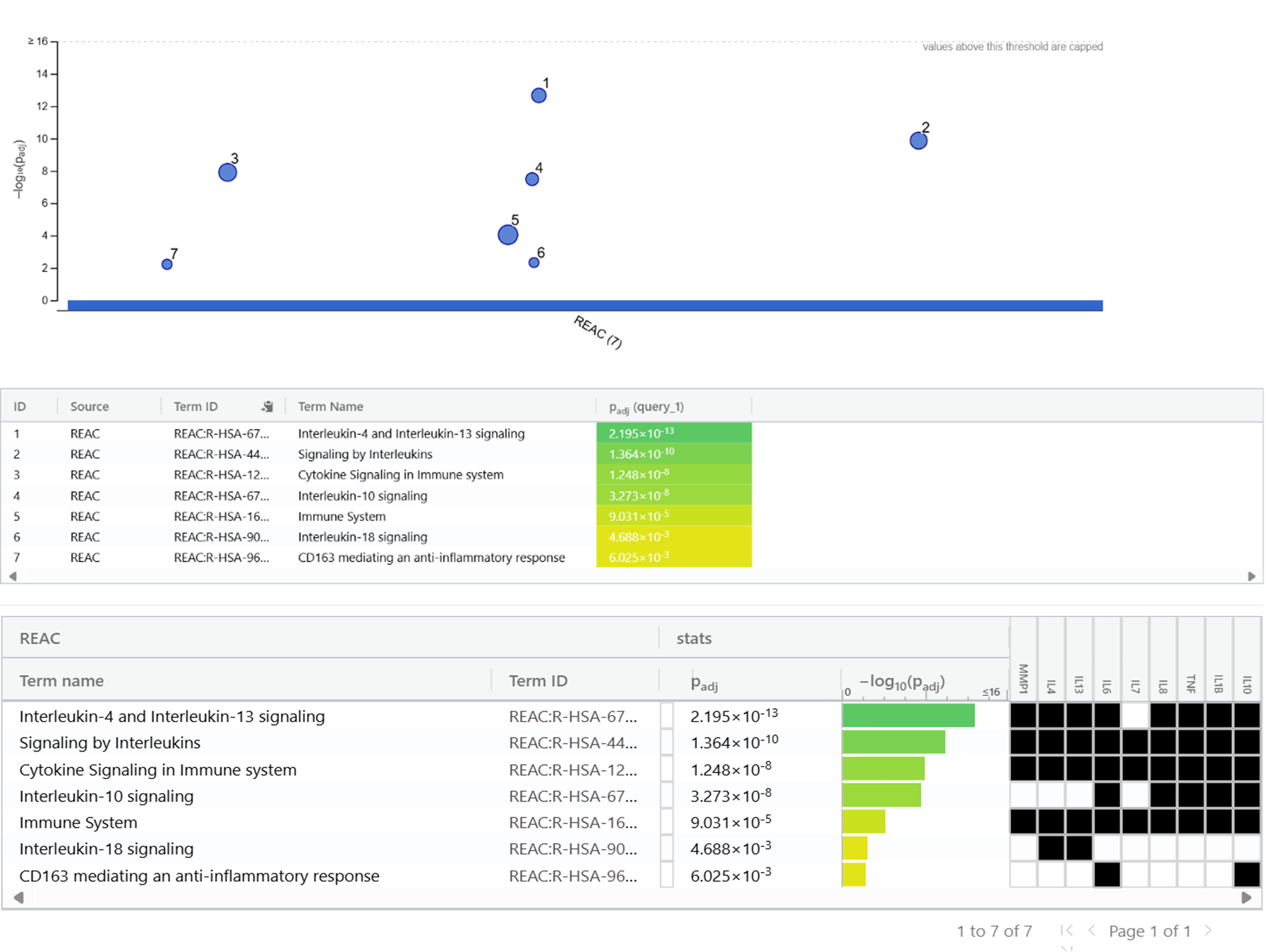

Supplement: Supplementary file 1 — Table S1: Inflammatory markers measured by Luminex assay. Figure S1: Assessment of Bronchodilator Response (BDR) Using Spirometry. This figure illustrates the spirometry procedure, where a patient performs inhalation and exhalation to measure lung function. The upper section represents the pre‐bronchodilator state, showing airway constriction and reduced airflow. The lower section represents the post‐bronchodilator state, where bronchodilator administration leads to airway relaxation and improved airflow. The graphs highlight Forced Expiratory Volume in 1 s (FEV1), a key parameter that measures the air breathed out forcefully within the first second after a deep breath. A significant improvement in FEV1 post‐bronchodilator is often used to confirm conditions like asthma. Figure S2: Directed Acyclic Graph (DAG) illustrating the potential confounders considered in the analysis of the relationship between bronchodilator response (BDR), asthma control, and serum cytokines/chemokines. The DAG identifies key variables, including age, sex, ethnicity, BMI z‐score, baseline lung function, country, season of inclusion, GINA step, and current smoking exposure, guiding appropriate adjustment in statistical models to minimize confounding bias. Figure S3: Overlap of high BDR classifications according to the two BDR definitions (>10% predicted and z‐score >0.78). All children identified by the >10% definition were also included in the z‐score group, with 8 additional children classified only by the z‐score. Table S2: (A) Demographic and clinical characteristics of children with high and low BDR according to the FEV1 z‐score definition. (B) Lung function, white blood cell counts, and atopic sensitization in children with high and low BDR according to the FEV1 z‐score definition. (C) Asthma medication use and treatment steps in children with high and low BDR according to the FEV1 z‐score definition. Table S3: Multi‐variate logistic regression model showing the association between BD [file PAI-37-e70392-s001.zip › Figure S8.png]
